# Identification and Characterization of the *APX* Gene Family and Its Expression Pattern under Phytohormone Treatment and Abiotic Stress in *Populus trichocarpa*

**DOI:** 10.3390/genes12030334

**Published:** 2021-02-25

**Authors:** Xue Leng, Hanzeng Wang, Shuang Zhang, Chunpu Qu, Chuanping Yang, Zhiru Xu, Guanjun Liu

**Affiliations:** 1 State Key Laboratory of Tree Genetics and Breeding, Northeast Forestry University, Harbin 150040, China; lengxue@nefu.edu.cn (X.L.); hzwang0@nefu.edu.cn (H.W.); cpqu@gzu.edu.cn (C.Q.); yangcp@nefu.edu.cn (C.Y.); xuzhiru2003@nefu.edu.cn (Z.X.); 2 College of Life Science, Northeast Forestry University, Harbin 150040, China; zhangshuang0609@nefu.edu.cn; 3 School of Forestry, Northeast Forestry University, Harbin 150040, China; 4 College of Forestry, Guizhou University, Guiyang 550025, China

**Keywords:** *APX*, *Populus trichocarpa*, RT-qPCR, phytohormone treatment, abiotic stress

## Abstract

Ascorbate peroxidase (APX) is a member of class I of the heme-containing peroxidase family. The enzyme plays important roles in scavenging reactive oxygen species for protection against oxidative damage and maintaining normal plant growth and development, as well as in biotic stress responses. In this study, we identified 11 *APX* genes in the *Populus trichocarpa* genome using bioinformatic methods. Phylogenetic analysis revealed that the PtrAPX proteins were classifiable into three clades and the members of each clade shared similar gene structures and motifs. The *PtrAPX* genes were distributed on six chromosomes and four segmental-duplicated gene pairs were identified. Promoter *cis*-elements analysis showed that the majority of *PtrAPX* genes contained a variety of phytohormone- and abiotic stress-related *cis*-elements. Tissue-specific expression profiles indicated that the *PtrAPX* genes primarily function in roots and leaves. Real-time quantitative PCR (RT-qPCR) analysis indicated that *PtrAPX* transcription was induced in response to drought, salinity, high ammonium concentration, and exogenous abscisic acid treatment. These results provide important information on the phylogenetic relationships and functions of the *APX* gene family in *P. trichocarpa*.

## 1. Introduction

On account of their immobility, plants are frequently subject to diverse environmental stresses, including biotic and abiotic stresses, such as insect attack, drought, chilling, and flooding [1]. Exposure to stress stimulates the production of reactive oxygen species (ROS), which induce a suite of stress responses in the plant. Accumulation of excessive ROS may cause injury to the plant, such as damage to cell membranes and organelles [2,3]. Ascorbate peroxidase (APX) is an important antioxidant enzyme involved in active oxygen metabolism in plants. The enzyme catalyzes the formation of O_2_ and H_2_O from the substrate hydrogen peroxide (H_2_O_2_) to protect the plant from oxidative damage caused by excessive H_2_O_2_ accumulation. Thus, APX plays a crucial role in redox homeostasis and the scavenging of ROS in plants [4,5].

Ascorbate peroxidase is a protease encoded by members of a polygenic family. The family is divided into four classes based on the subcellular localization of the protein, namely the cytosol, peroxisomes, chloroplasts, and mitochondria [5,6]. In *Arabidopsis thalian*a, eight members of the *AtAPX* gene family have been identified, which encode three cytosolic (*AtAPX1*, *-2*, and *-6*), three peroxisomal (*AtAPX3*, -*4*, and -*5*), and two chloroplastic (*AtsAPX* and *AttAPX*) proteins [7,8], of which *AtsAPX* binds to both the chloroplast matrix and mitochondria [9]. In rice (*Oryza sativa*), eight APX genes have been identified, including two cytosolic isozymes (*OsAPX1* and *-2*), two peroxisomal isozymes (*OsAPX3* and -*4*), three chloroplastic isozymes (*OsAPX5*, -*7*, and *-8*), and one mitochondrial isozyme (*OsAPX6*) [10,11,12]. The function of an APX protein varies with its subcellular localization. For instance, APX localized in mitochondria is mainly involved in scavenging H_2_O_2_ produced by β-oxidation of fatty acids [13]. Cytoplasmic *OsAPX2* plays important roles in chloroplast protection and H_2_O_2_ homeostasis [14]. Silencing of *OsAPX4*, which encodes a peroxisomal protein, results in a premature aging phenotype in rice [10]. The same phenotypic response is observed in other plants species, such as *Arabidopsis* [15] and *Chenopodium album* [16].

Ascorbate peroxidase genes play important roles in plant growth, development, and response to environmental stress. The *Arabidopsis apx1* mutant grows slowly and is sensitive to oxidative stress in comparison with the wild type (ecotype Col-0) [2,17,18]. The *apx1 tapx* double mutant of *Arabidopsis* shows substantial accumulation of anthocyanins and delayed flowering time under light stress [8,19]. The ROS content is increased and the germination frequency is reduced in seeds of the *Arabidopsis apx6 mutant* [20]. Loss-of-function of *OsAPX4* results in a premature senescence phenotype in leaves of rice [21]. The rice *apx2* mutant exhibits semi-dwarf seedlings, yellow-green leaves, and sterile seeds [22]. Expression of a rice cytosolic *OsAPX* gene in transgenic *Arabidopsis* elevates salt tolerance [23]. Drought stress tolerance is enhanced by over-expression of a peroxisomal ascorbate peroxidase (*SbpAPX*) of *Salicornia brachiata* in tobacco (*Nicotiana tabacum*) [24]. The expression level of the majority of *APX* genes is up-regulated in response to abscisic acid (ABA) treatment in maize (*Zea mays)* [25]. However, in contrast to studies of *APX* genes in herbaceous plant species, there is a paucity of research on genome-wide identification and expression pattern analysis of the *APX* gene family in woody plants.

*Populus* is characterized by its ease of vegetative propagation and rapid growth rate, and considerable genomic information is publicly available [26,27]. As a model species for woody plants, *Populus trichocarpa* is widely used in forest tree molecular breeding and resistance breeding research. The *PpAPX* gene in *Populus tomentosa* has been cloned and it has been found that overexpression of *PpAPX* increases drought and salt tolerance in transgenic tobacco [28,29]. *PtomtAPX* and *PtosAPX* located in mitochondria of *Populus tomentosa* Carr were identified. *PtosAPX* is dual targeted to both chloroplast and mitochondria, while *PtomtAPX* is specifically targeted to mitochondria and plays an important role in maintaining the redox balance [30]. As an important component of the ROS antioxidant system, the molecular characteristics of *PtrAPX* genes and their functions require detailed investigation. In this study, we identified 11 *APX* genes in the *P. trichocarpa* genome. We analyzed the chromosomal location, gene structure, phylogenetic relationships, physicochemical properties, and expression patterns of members of the *PtrAPX* gene family in response to abiotic stress and exogenous ABA treatment. The results provide a foundation for future exploration of the molecular mechanisms of APXs in poplar exposed to environmental stresses.

## 2. Materials and Methods

### 2.1. Identification of APX Genes in P. trichocarpa Genome

The amino acid sequences of the eight identified *AtAPX* genes were downloaded from The Arabidopsis Information Resource database (https://www.arabidopsis.org/, accessed on 2 September 2020). We used the protein family database Pfam (http://pfam.xfam.org/, accessed on 3 September 2020) to search for the hidden Markov model (HMM) profile of the *APX* gene family (protein family ID: PF06200) based on an expected value (*E*-value) cutoff of 1× 10^-5^ in HMMER 3.3 (http://hmmer.org, accessed on 3 September 2020) to find *APX* genes in the *P. trichocarpa* genome and then remove the redundancy with some specifications. All candidate *PtrAPX* genes were assessed for presence of the conserved Haem-peroxidase domain (IPR002016) using the InterProScan database (http://www.ebi.ac.uk/interpro/, accessed on 3 September 2020) and peroxidase (PF00141) in the pfam database (http://pfam.xfam.org/, accessed on 3 September 2020). The subcellular localization of the PtrAPX proteins was predicted using WoLF PSORT (https://www.genscript.com/wolf-psort.html, accessed on 5 September 2020). Physicochemical properties of the PtrAPX amino acid sequences, such as theoretical isoelectric point, molecular weight, number of amino acids, aliphatic index, and grand average of hydropathicity (GRAVY) index, were estimated using the ProtParam tool of the Expasy (https://web.expasy.org/protparam/, accessed on 5 September 2020) online portal. Transmembrane analysis was conducted using the TMHMM Server v2.0 (https://www.cbs.dtu.dk/services/TMHMM/, accessed on 5 September 2020) online database.

### 2.2. Gene Structure and Conserved Motif Analysis

The exon–intron structure of the PtrAPX gene family members was analyzed using the Gene Structure Display Server (GSDS 2.0 http://gsds.cbi.pku.edu.cn/, accessed on 6 September 2020). The conserved motifs of the PtrAPX proteins were identified using the MEME Suite 5.1.1 (MEME http://meme-suite.org/tools/meme, accessed on 6 September 2020) online tools.

### 2.3. Chromosomal Location, Duplication Analysis, and Ka/Ks Calculation

The chromosomal location of *PtrAPX* genes in the *P. trichocarpa* genome was determined using the PopGenIE v3: The Populus Genome Integrative Explorer database (http://popgenie.org/, accessed on 8 September 2020). A schematic representation of the chromosomal locations was generated using the MapGene2Chromosome v2 (http://mg2c.iask.in/mg2c_v2.0/, accessed on 8 September 2020) online tool. For Ka/Ks calculation, the coding sequences of paralogous gene pairs performed multiple alignment using ClustalW, and then, the values of Ka and Ks were calculated using Synonymous and NonSynonymous Substitutions in DnaSP software (version 6.12.03) [31]. The divergence time (T) was calculated as T = Ks/2 × 6.1 × 10^−9^ Mya [32].

### 2.4. Promoter cis-Element Analysis

The promoter sequence (2 kb upstream of the initiation codon) of each *PtrAPX* gene family member was downloaded from the Phytozome v12.1 database using the corresponding *PtrAPX* gene ID. The PlantCARE online database (http://bioinformatics.psb.ugent.be/webtools/plantcare/html/, accessed on 9 September 2020) was used to identify the abiotic stress- and phytohormone-related *cis*-elements in the *PtrAPX* promoters. IBS: Illustrator for Biological Sequences software was used to visualize the results.

### 2.5. Multiple Sequence Alignment and Phylogenetic Analysis

The PtrAPX protein sequences were aligned using Clustal X 2.0 [33] and Bioedit 7.2.5 software. MEGA 7.0 [34] was used to construct a phylogenetic tree (with 1000 bootstrap replications) of the APX gene family in *P. trichocarpa*, *Arabidopsis*, and rice using the neighbor-joining (NJ) method.

### 2.6. Prediction of the Secondary and Tertiary Structure of Proteins

We predicted the secondary structure of the PtrAPX proteins using the NPSA (https://npsa-prabi.ibcp.fr/cgi-bin/npsa_automat.pl?page=npsa_sopma.html, accessed on 12 September 2020) online software. The tertiary structure of PtrAPX proteins was predicted using the SWISS-MODEL database (https://swiss model.expasy.org/, accessed on 2 November 2020).

### 2.7. Tissue-Specific Expression Analysis

Visualized images of the tissue-specific expression pattern for each *PtrAPX* gene were downloaded from the PopGenIE v3 database (http://popgenie.org/, accessed on 12 September 2020) using the gene ID [35]. Then we used three-week-old in vivo *P. trichocarpa* to verify the expression level of each *PtrAPX* gene in roots and leaves using RT-qPCR. The specific primers are listed in Supplementary Table S1.

### 2.8. Plant Materials, Abiotic Stress, and Phytohormone Treatment

In vivo *P. trichocarpa* (genotype Nisqually-1) was cultured in a tissue culture laboratory under a 16/8 h (light/dark) photoperiod with light intensity of 50 µmol photons m^−2^ s^−1^ at 23–25 °C. Wild-type *P. trichocarpa* seedlings were cultured on a woody plant medium (WPM; pH 5.8) for 3 weeks to use for abiotic stress and phytohormone treatment. For abiotic stress treatment, consistently growing 3-week-old *P. trichocarpa* were transferred to a WPM supplemented with 7% PEG6000, 150 mM NaCl, or 10 mM (NH_4_)_2_SO_4_ (high ammonium: NH_4_^+^). For phytohormone treatment, wild-type *P. trichocarpa* was transferred to a WPM supplemented with 200 µM ABA. Each abiotic stress and phytohormone treatment was applied for 0, 3, 6, 12, and 24 h, of which the 3 and 6 h time points served as an early response, whereas the 12 and 24 h time points served as a later response. Nontreated seedlings of *P. trichocarpa* served as the control. Fresh roots and leaves from plants in each stress and phytohormone treatment were sampled at the corresponding time points, immediately frozen in liquid nitrogen, and stored at −80 °C until use. Each sample consisted of three biological replicates.

### 2.9. RNA Extraction and RT-qPCR Analysis

Total RNA was extracted from different tissues using pBIOZOL Plant Total RNA Extraction Reagent (BioFlux, Hangzhou, China) according to the protocol; then the extracted total RNA was treated with gDNA Eraser to remove genomic DNA as following the reaction system (5× g Eraser Buffer 2 μL, gDNA Eraser 1 μL, total RNA 1 µg) (Takara Bio, Dalian, China). Next, total RNA (1 µg) was reverse transcribed using the PrimeScriptTM RT kit (Takara Bio, Dalian, China) to obtain cDNA as following reaction system (PrimerScript RT Enzyme Mix I 1 μL, RT Primer Mix 1 μL, 5× PrimerScript Buffer 4 μL, total RNA 1 µg). The quantitative real-time PCR used an UltraSYBR Mixture (Low ROX) (CWBIO) with three biological replicates, and the detailed procedure referred to the manufacturer’s instructions. The RT-qPCR reaction was performed by qTOWER 3G Cycler (Analytik Jena, Germany) and the relative expression level analysis was calculated using 2^−△△CT^ method [36,37]. We selected the *UBQ7* gene as an internal reference gene [38,39]. All *PtrAPX* specific primers for RT-qPCR and *UBQ7* gene primers are listed in Supplementary Table S1.

### 2.10. Gene Ontology of P. trichocarpa APXs

The gene ontology (GO) annotation of *PtrAPX* genes was conducted using Blast2GO v5.2 software. First, we uploaded all protein sequences to Blast2GO and blasted the sequences in the NCBI online database. After mapping and annotation, the GO results and visualized images were downloaded. All procedures were conducted using the default parameters.

### 2.11. Statistical Analysis

Student’s *t* test was performed using SPSS (version, IBM, Chicago, IL, USA) software to determine significance. Significance was defined as * *p* < 0.05, ** *p* <0.01. The detailed statistical analysis is listed in Supplementary Table S2.

## 3. Results

### 3.1. Identification of APX Genes in the P. trichocarpa Genome

Eight members of the *APX* gene family have been identified previously in the *Arabidopsis* genome, consisting of *AtAPX1–6*, *AtsAPX (*stromal *APX)*, and *AttAPX (*thylakoid membrane- bound *APX*) [7,8,9]. To identify all *APX* genes in the *P. trichocarpa* genome, we used the eight *AtAPX* protein sequences for a BLAST search to detect homologs in the *P. trichocarpa* genome. Eleven candidate *APX* gene family members (hereafter *PtrAPX*) were identified in the *P. trichocarpa* genome. The 11 *PtrAPX* genes all possessed the ascorbic acid peroxidase domain (protein ID: IPR002016) as predicted with the InterProScan database. The *PtrAPX* genes were designated *PtrAPX1* to *PtrAPX11* based on the chromosomal location. The protein length and molecular weight ranged from 96 to 486 aa and 10,226.73 to 53,657.88 Da, respectively. The aliphatic index ranged from 72.6 to 92.5. The GRAVY score ranged from −0.491 to 0.255. PtrAPX6 possessed the maximum aliphatic index and GRAVY score, and was the only gene to show a positive GRAVY score. Prediction of the subcellular localization of the PtrAPX proteins using WoLF PSORT revealed that PtrAPX2, -3, -6, -7, -9, -10, and -11 were predicted to be cytoplasmic, PtrAPX1 and 8 were predicted to be localized to the chloroplast stroma and chloroplast thylakoid membrane, respectively, and PtrAPX4 and -5 were predicted to be localized to the chloroplast. Details for the *PrtAPX* genes are presented in Table 1.

### 3.2. Phylogenetic Analysis

**Figure 1:**
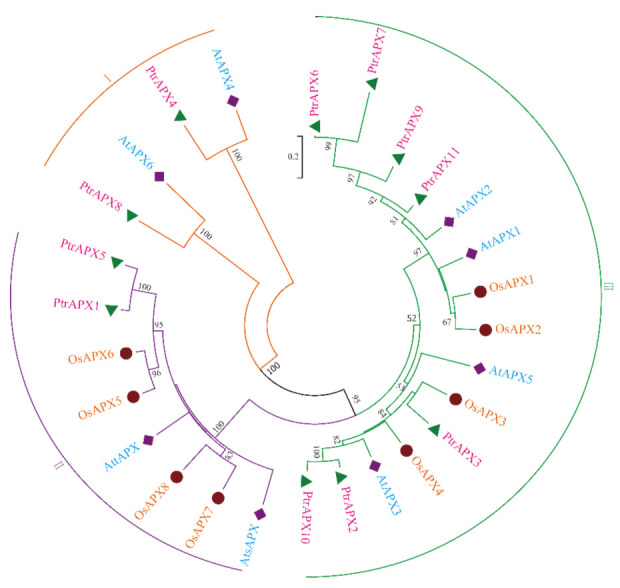
Phylogenetic analysis of APX proteins from *Populus trichocarpa*, *Arabidopsis thaliana*, and *Oryza sativa.* A multiple alignment of the full-length amino acid sequences was generated using Clustal X 2.0. The unrooted phylogenetic tree was constructed using the neighbor-joining method with 1000 bootstrap replicates using MEGA 7.0. Different species are represented by different colors. The APX proteins are classified into three clades (I, II, and III) based on their subcellular localization.

To evaluate the evolutionary relationships among plant *APX* genes, an unrooted NJ tree was constructed derived from *Arabidopsis*, rice, and *P. trichocarpa* APX protein sequences. The proteins were resolved into three clades (Figure 1). Previous research has shown that the classification of *APX* genes is consistent with the subcellular localization of the protein [5,40]. Thus, based on the predicted subcellular localization of the PtrAPX proteins, the three clades of the unrooted NJ tree were designated I (cytoplasmic), II (chloroplastic or mitochondrial), and III (chloroplastic). The majority of PtrAPX proteins were classified in clade III, PtrAPX1 and five were placed in clade II, and PtrAPX4 and -8 were classified in clade I. Rice is a monocotyledon and OsAPX proteins were predominantly clustered together. The majority of PtrAPX family members were clustered with *Arabidopsis* proteins, which indicated that PtrAPX genes were phylogenetically more closely related to those of *Arabidopsis* than to those of rice. The accession numbers and amino acid sequences of *P. trichocarpa*, *Arabidopsis* and rice are provided in Supplemental File 1.

### 3.3. Gene Structure and Conserved Motifs of PtrAPX Genes

**Figure 2:**
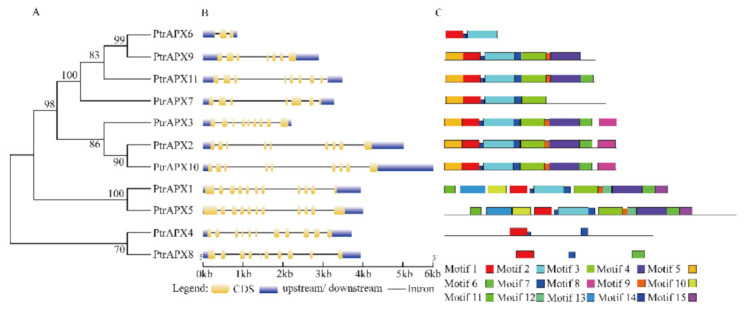
Phylogenetic tree, gene structure, and conserved motif analysis of the 11 *PtrAPX* genes. (**A**) A multiple alignment of the full-length PtrAPX protein sequences was generated using Clustal X 2.0. The phylogenetic tree was constructed using the neighbor-joining method with 1000 bootstrap replicates using MEGA 7.0. (**B**) Intron–extron structure of the corresponding *PtrAPX* genes. (**C**) Conserved motifs of *PtrAPX* genes predicted by the MEME tool. The motif numbers 1–15 are represented by boxes of different colors.

To further explore the phylogenetic relationships among PtrAPX proteins, an NJ tree was constructed from a multiple alignment of full-length PtrAPX protein sequences. We identified four paralogous pairs (*PtrAPX1* and -*5*, *PtrAPX2* and -*10*, *PtrAPX4* and -*8*, and *PtrAPX6* and -*9*), all of which received strong bootstrap support (>70%; Figure 2A). Analysis of the exon–intron organization revealed that the homologs showed similar numbers of exons and introns. For example, *PtrAPX2* and its homolog, *PtrAPX10*, contained eight introns, the same number as *PtrAPX1* and *5*, and *PtrAPX4* and -*8*. *PtrAPX6* contained the fewest introns (Figure 2B). The MEME database was used to analyze conserved motifs in the *PtrAPX* genes (Supplementary Table S3). All *PtrAPX* genes contained motif 1 and nine *PtrAPX* genes possessed motif 3. The paralogous pairs contained similar motifs, such as P*trAPX2* and -*10*. *PtrAPX1* and -*5* contained -13 motifs, whereas *PtrAPX4*, -*6*, and -*8* contained three motifs (Figure 2C).

### 3.4. Chromosomal Location and Duplications of PtrAPX Genes

**Figure 3:**
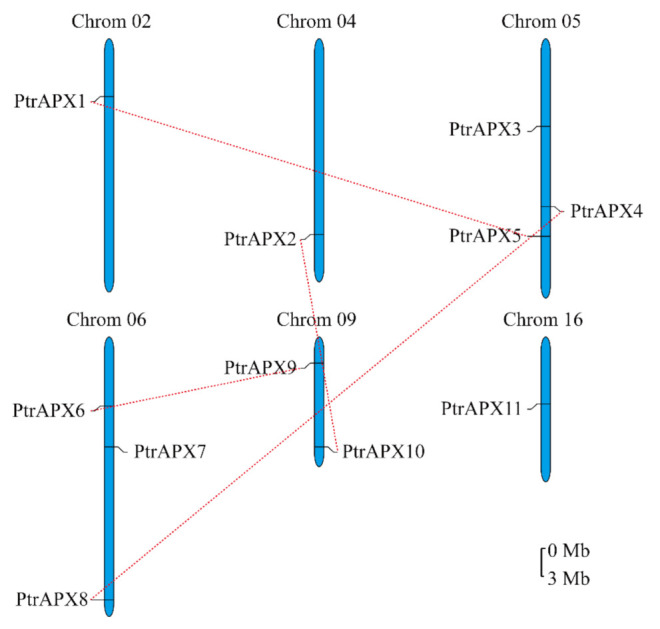
Chromosomal location and gene duplication of *PtrAPX* genes. The 11 *PtrAPX* genes were distributed on six chromosomes in the *P. trichocarpa* genome. Red dotted lines show the links between duplicated gene pairs. Scale bar = 3Mb.

The chromosomal locations of *PtrAPX* genes were physically mapped on the 19 chromosomes of *P. trichocarpa*. The 11 *PtrAPX* genes were distributed on six chromosomes, namely chromosomes 02, 04, 05, 06, 09, and 16 (Figure 3). Chromosomes 05 and 06 each carried three *PtrAPX* genes. *PtrAPX1*, *2*, and *11* were located on chromosomes 02, 04, and 16, respectively. *PtrAPX9* and *10* were located on chromosome 09. All genes except *PtrAPX7* were located in a duplicated chromosomal region. To better understand the evolutionary constraints associated with the *PtrAPX* genes the Ka/Ks values were calculated. Detailed information on the Ka/Ks values of the *PtrAPX* genes is listed in Table 2. The Ka/Ks values of the *PtrAPX1*–*PtrAPX5, PtrAPX2*–*PtrAPX10* and *PtrAPX4*–*PtrAPX8* paralogous pairs were less than 1, which indicated that the evolution of the majority of paralogous pairs was under purifying selection. Therefore, the gene pairs may have the same functions. The Ka/Ks value of the *PtrAPX6*–*PtrAPX9* paralogous pair was 1.22, which suggested that these genes underwent positive selection and may be involved in different functions.

### 3.5. Promoter cis-Element Analysis

**Figure 4:**
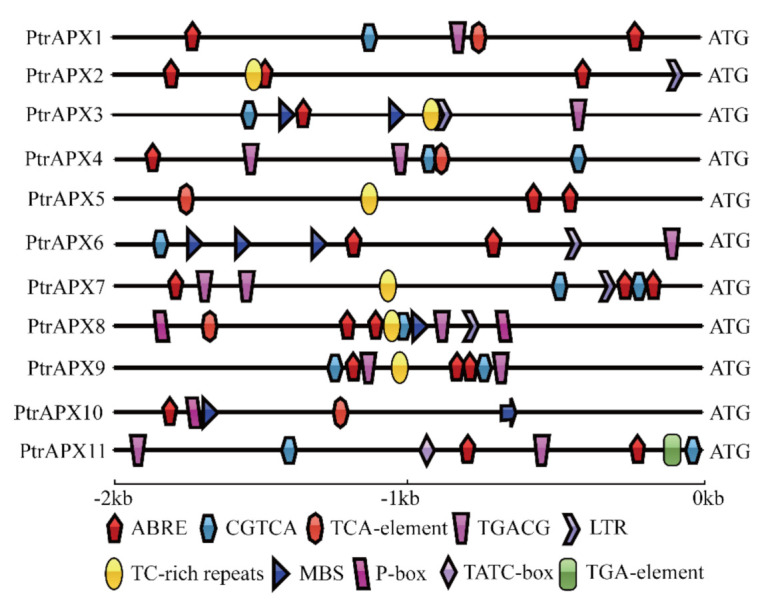
*cis*-Acting regulatory elements of *PtrAPX* genes involved in response to phytohormones and abiotic stress. Different *cis*-elements are indicated by symbols of different shapes.

Promoter *cis*-elements function as transcription factor binding sites and play an important role in transcriptional regulation in response to abiotic stress and phytohormone treatment [41]. We identified putative abiotic stress- and phytohormone-related *cis*-elements in the promoter region of *PtrAPX* genes to explore the possible regulatory mechanisms of the genes. Ten types of abiotic stress- and phytohormone-related *cis*-elements were identified (Figure 4). With regard to phytohormone-related *cis*-elements, all *PtrAPX* genes contained the ABRE element (involved in ABA responsiveness), which indicated that *PtrAPX* genes may play a pivotal role in ABA response. *PtrAPX8* and *10* contained the P-box (a gibberellin-responsive element). *PtrAPX1*, *4*, *5*, *8*, and *10* possessed the TCA element (a *cis*-acting element involved in salicylic acid responsiveness). Only *PtrAPX11* contained an additional gibberellin-responsive element (TATC-box) and the TGA-element (an auxin-responsive element). Interestingly, eight *PtrAPX* genes possessed CGTCA and TGACG motifs, which are both involved in methyl jasmonate responsiveness. With regard to abiotic stress-related cis-elements, all but *PtrAPX1*, *3*, *6*, *10*, and *11* contained TC-rich repeats (*cis*-acting elements involved in defense and stress responsiveness). These four genes each contained the LTR element (involved in low-temperature responsiveness) and MYB-binding site (MBS; involved in drought-inducibility). Importantly, *PtrAPX6* contained three MBS cis-elements in the promoter region, which indicated that *PtrAPX6* may participate in the response to drought stress. It was notable that *PtrAPX3*, *6*, and *8* contained all abiotic stress-related *cis*-elements, which indicated that these three genes may play an important role in abiotic stress response.

### 3.6. Prediction of the Secondary and Tertiary Structure of APX Proteins

**Figure 5:**
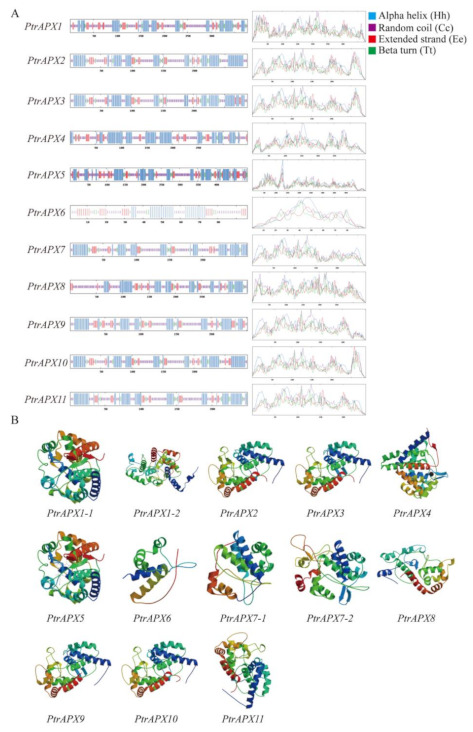
Secondary and tertiary structures of PtrAPX proteins. (**A**) Secondary structure of PtrAPX proteins. Motifs are represented by different colors: blue, α-helix; purple, random coil; red, extended strand; green, β-turn. (**B**) Tertiary structure of PtrAPX proteins.

Protein is the basic unit of life activities and its structure determines its function. The study of protein structure is helpful to gain a thorough understanding of its function [42]. The secondary and tertiary structures of APX proteins was predicted using the Expaxy online database. The secondary structure of the PtrAPX proteins were classified into four structural patterns: α-helix, β-turn, extended strand, and random coil (Figure 5A). In the PtrAPX proteins, the random coil contained the highest number of amino acids, followed by the α-helix (Supplementary Table S4). With regard to the tertiary structure, a variety of structures were predicted. PtrAPX1 and 7 contained two types of tertiary structure (Figure 5B), which indicated that the PtrAPX proteins perform diverse functions. The templates and PDB files of each PtrAPXs proteins are provided in Supplementary File 2.

### 3.7. GO Annotation

**Figure 6:**
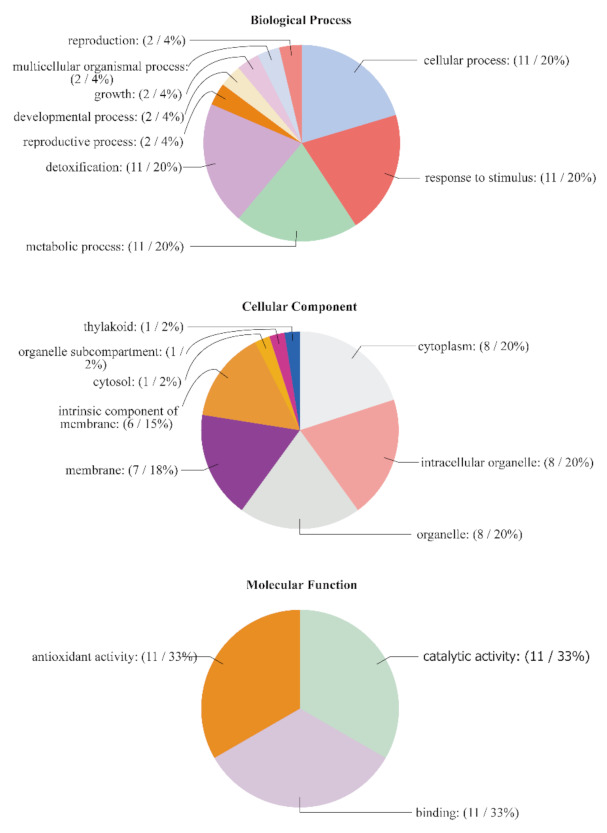
Gene ontology analysis of the *PtrAPX* gene family. The gene ontologies were predicted for the categories of biological processes, molecular functions, and cellular components.

The GO annotations were classified into three categories: cellular components, molecular functions, and biological processes. Detailed information on the GO annotation of the PtrAPX genes is provided in Supplemental Table S5. For cellular components, eight PtrAPX proteins were localized in the cytoplasm, intracellular organelles and organelles. Seven PtrAPX proteins were localized in membranes, six in intrinsic components of membranes, and one each in the thylakoids, organelle subcompartment, and cytosol. With regard to molecular functions, all *PtrAPX* gene family members participated in antioxidant activity, catalytic activity, and binding. The *PtrAPX* genes participated in a variety of biological processes; for instance, two *PtrAPX* genes are involved in reproduction, multicellular organismal processes, growth, and developmental and reproductive processes. All *PtrAPX* genes participated in detoxification, cellular processes, and response to stimulus processes (Figure 6). These results suggested that *PtrAPX* genes played an important role in plant development and growth as well as response to environmental stresses.

### 3.8. Tissue-Specific Expression Profiles of PtrAPX Genes

**Figure 7:**
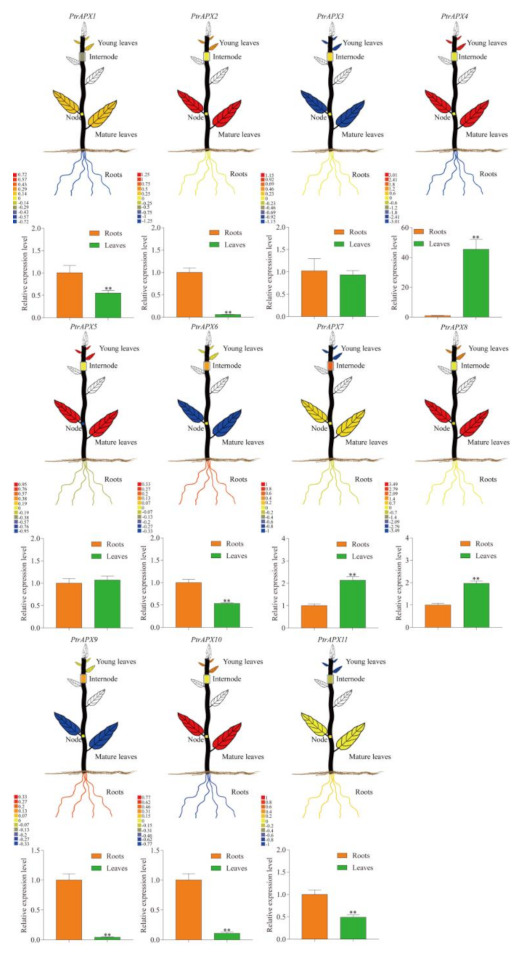
Tissue-specific expression profiles of *PtrAPX* genes. The visual image for each *PtrAPX* gene in *P. trichocarpa* was obtained from PopGenIE [43]. Error bars represent the standard deviations from three biological replicates. Asterisks indicate stress treatment groups that showed a significant difference in transcript abundance compared with the control group (** *p* < 0.01).

To analyze the possible roles of *PtrAPX* genes in the developmental processes of *P. trichocarpa*, visualized images were downloaded for different tissues or organs, consisting of roots, nodes, internodes, mature leaves, and young leaves, from the PopGenIE v3 database (accession number: GSE6422) [43]. The genes *PtrAPX1*, *4*, and *9* exhibited a high relative expression level and *PtrAPX6* and *9* showed a low expression level in roots (Figure 7). Five *PtrAPX* genes showed a high expression level in leaves: *PtrAPX2*, *4*, *5*, *8*, and *10*. The relative expression levels of *PtrAPX3*, *6*, and *9* were low. Four *PtrAPX* genes expressed in internodes and three genes expressed in nodes showed a high relative expression level. Next, we further verified the validation of previous microarray data using RT-qPCR. As shown in Figure 7, the results of RT-qPCR were generally consistency with the microarray analysis and had very distinct tissue-specific expression pattern in roots and leaves (Figure 7). It is worth noting that *PtrAPX1*. -*2* and -*10* exhibited different expression patterns between the RT-qPCR analysis and microarray analysis, which might be caused by the differences of their experimental materials, such as conditions, sample collection times, etc. [44].

### 3.9. RT-qPCR Analysis of PtrAPXs Response to Drought and NaCl Stress

**Figure 8:**
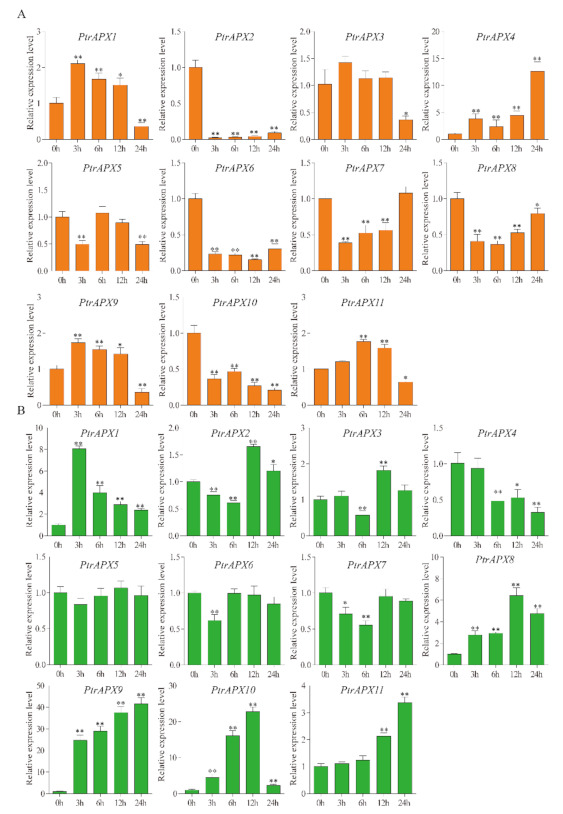
Relative expression level of *PtrAPX* genes under drought stress estimated by RT-qPCR. The expression pattern of *PtrAPX* genes in roots (**A**) and leaves (**B**) is shown. Error bars represent the standard deviations from three biological replicates. The x-axis represents time points after PEG treatment. Asterisks indicate stress treatment groups that showed a significant difference in transcript abundance compared with the control group (* 0.01< *p* < 0.05, ** *p* < 0.01).

During growth and development, poplar plants are frequently subjected to a variety of abiotic stresses, including drought and high salinity [45,46]. Many genes are induced and contribute to resistance when the plant is exposed to these environmental stresses [47]. Therefore, the relative expression level of *PtrAPX* genes was detected in response to drought and salinity treatment in *P. trichocarpa*. Under drought stress, eight *PtrAPX* genes were induced by treatment with 7% PEG6000, and expression of *PtrAPX4*, *6*, and *7* was suppressed at several time points. *PtrAPX1* and *9* were significantly upregulated at all time points (*p* < 0.01). It was notable that *PtrAPX9* was gradually upregulated in leaves at successive time points and expression peaked at 24 h, when the gene was up-regulated by approximately 40-fold compared with leaves of nontreated plants. In roots, *PtrAPX1*, *4*, *9*, and *11* were induced, whereas *PtrAPX2*, *6*, *8*, and *10* were suppressed at all time points. *PtrAPX7* was rapidly downregulated at 3, 6, and 12 h, and thereafter returned to the normal expression level. The genes *PtrAPX1*, *9*, and *11* were induced in roots and leaves in response to drought stress (Figure 8).

**Figure 9:**
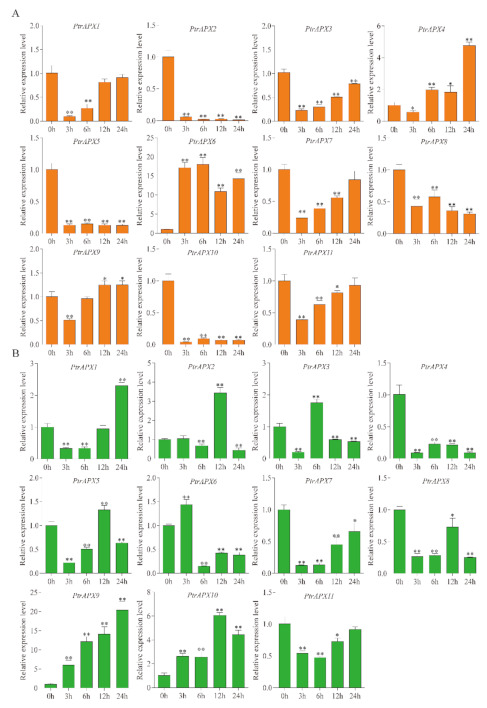
Relative expression level of *PtrAPX* genes under 150 mM NaCl treatment estimated by RT-qPCR. The expression pattern of *PtrAPX* genes in roots (**A**) and leaves (**B**) is shown. Error bars represent the standard deviations from three biological replicates. The x-axis represents time points after NaCl treatment. Asterisks indicate stress treatment groups that showed a significant difference in transcript abundance compared with the control group (* 0.01< *p* < 0.05, ** *p* < 0.01).

The expression profiles of *PtrAPX* genes under 150 mM NaCl stress were also analyzed. Seven *PtrAPX* genes were induced and the remainder were suppressed in leaves (Figure 9). *PtrAPX4*, *7*, and *8* were significantly downregulated at all time points. *PtrAPX6* was rapidly induced at 3 h and suppressed at 6, 12, and 24 h. *PtrAPX11* was strongly down-regulated in the early response period and thereafter returned to the normal expression level at 24 h. The majority of *PtrAPX* genes were suppressed in roots, except for *PtrAPX4*, *6*, and *9*, which were induced. Both *PtrAPX4* and *9* were significantly downregulated at 3 h and upregulated at 12 and 24 h, which indicated that these two *PtrAPX* genes may represent late-response genes. Notably, *PtrAPX6* was strongly up-regulated at all time points (*p* < 0.01).

### 3.10. RT-qPCR Analysis of PtrAPX Genes under ABA Treatment

**Figure 10:**
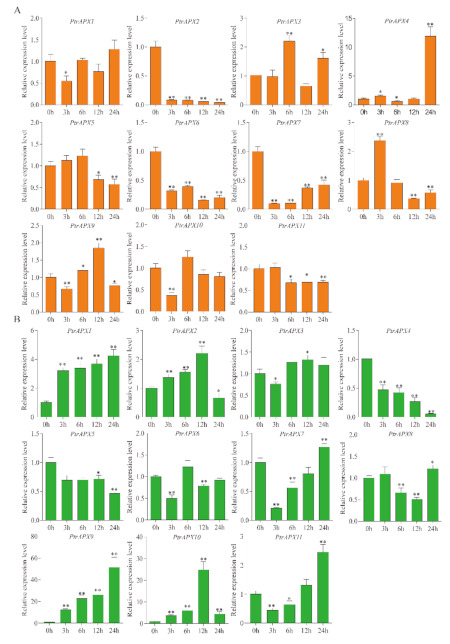
Relative expression level of *PtrAPX* genes under 200 μM abscisic acid (ABA) treatment estimated by RT-qPCR. The expression pattern of *PtrAPX* genes in roots (**A**) and leaves (**B**) is shown. Error bars represent the standard deviations from three biological replicates. The x-axis represents time points after ABA treatment. Asterisks indicate stress treatment groups that showed a significant difference in transcript abundance compared with the control group (* 0.01< *p* < 0.05, ** *p* < 0.01).

Expression of the *PtrAPX* genes in response to 200 μM ABA treatment was investigated using RT-qPCR analysis. Half of the *PtrAPX* genes were induced, whereas *PtrAPX4* and *5* were significantly suppressed at all time points in leaves (*p* < 0.01; Figure 10). *PtrAPX7* and *11* showed similar expression patterns, i.e., down-regulation at 3 and 6 h, and upregulation at 12 and 24 h. *PtrAPX9* was rapidly induced at 3 h, then was gradually up-regulated at subsequent time points and expression peaked at 24 h, at which point it was up-regulated by approximately 50-fold compared with the control. Four *PtrAPX* genes were induced in roots. Interestingly, the *PtrAPX4* expression level showed no significant change at 3, 6, and 12 h (*p* > 0.05), but was rapidly up-regulated by approximately 12-fold at 24 h. *PtrAPX1* and *10* were significantly downregulated at 3 h and returned to the normal expression level at 6, 12, and 24 h. *PtrAPX2*, *6*, and *7* were suppressed at all time points in roots. *PtrAPX3*, *8*, and *9* were induced, whereas *PtrAPX5*, *6*, and *7* were suppressed, in roots and leaves under ABA treatment.

### 3.11. RT-qPCR Analysis of PtrAPX Genes under Ammonium Treatment

**Figure 11:**
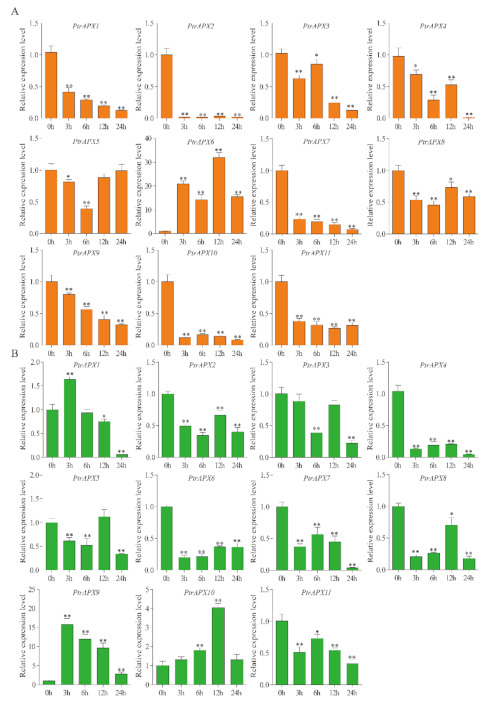
Relative expression level of *PtrAPX* genes under 10 mM (NH_4_)_2_SO_4_ treatment estimated by RT-qPCR. The expression pattern of *PtrAPX* genes in roots (**A**) and leaves (**B**) is shown. Error bars represent the standard deviations from three biological replicates. The x-axis represents time points after (NH_4_)_2_SO_4_ treatment. Asterisks indicate stress treatment groups that showed a significant difference in transcript abundance compared with the control group (* 0.01< *p* < 0.05, ** *p* < 0.01).

Almost all *PtrAPX* genes, except *PtrAPX1*, *9*, and *10*, were suppressed in leaves under NH_4_^+^ treatment. *PtrAPX9* was dramatically up-regulated at all time points. *PtrAPX10* showed no significant change in expression in roots and gradual up-regulation with time in leaves, which peaked at 12 h (approximately four-fold increase) and thereafter declined. *PtrAPX2*, -*4*, -*7*, -*8*, and -*11* were significantly down-regulated at all time points in leaves (Figure 11). In roots, only *PtrAPX6* was induced and showed a high expression level at all time points, whereas the remaining *PtrAPX* genes were all suppressed. Among these down-regulated genes, *PtrAPX1*, -*2*, -*3*, -*4*, -*7*, -*8*, -*9*, -*10*, and -*11* were significantly down-regulated at all time points (*p* < 0.05). *PtrAPX2*, -*3*, -*4*, -*5*, -*7*, and -*8* were suppressed in roots and leaves (Figure 11).

## 4. Discussion

Ascorbate peroxidase genes play an important role in the detoxification of photoproduced H_2_O_2_ and diverse abiotic stresses, such as drought, low temperature, high salinity, and iron stress [48]. *APX* genes have been characterized in, for example, *Arabidopsis* [8,49], rice [11,50], tomato (*Solanum lycopersicum*) [51], and cotton (*Gossypium hirsutum*) [52]. Limited information on the *APX* gene family is available for the model woody plant, *Populus trichocarpa*. In the present study, we identified 11 *APX* gene family members in the *P. trichocarpa* genome, each of which contained the conserved ascorbic acid peroxidase domain. The number of *PtrAPX* genes is notably higher than that reported for *Arabidopsis* and rice (which both possess eight members). This difference may suggest that *APX* genes perform more complex functions in woody plants.

Previous studies have identified seven APX genes in *Populus trichocarpa* [53]*,* In this study, 11 *PtrAPX* genes have been identified encoding seven cytosolic isoforms, and four chloroplastic isoforms. Among the *PtrAPXs* genes located in chloroplast, *PtrAPX1* and *PtrAPX5* are dual-targeted to chloroplasts and/or mitochondria, consistent with the findings that *PtosAPX* and *AtsAPX* are dual-targeted to these two organelles [9,30].

Integration and realignment of gene fragments may lead to an increase or decrease in the number of exons or introns [54]. In the current study, the majority of *PtrAPX* members of class I contained eight introns, except for *PtrAPX6*, *7*, and *9*, which possessed two, six, and six introns, respectively (Figure 2). It is notable that *PtrAPX6* and *9* were paralogous gene pairs. These results indicated that a number of gene evolutionary events accrued in these genes and led to the increase or decrease in intron number, and may have resulted in functional differences. This phenomenon is even observed among homologous genes.

It is generally accepted that promoter *cis*-elements play an important role in the transcriptional regulation that controls diverse biological processes, such as responses to exogenous phytohormone treatment and abiotic stress [55]. In the present report (Figure 4), we identified numerous phytohormone- and abiotic stress-related *cis*-elements in the promoter region of *PtrAPX* genes, including ABRE, CGTCA-motif, TCA-element, LTR, TC-rich repeat, TGA-element, TATC-box, P-box, and MBS *cis*-elements. Each *PtrAPX* gene contained at least one phytohormone- and one abiotic stress-related *cis*-element, which indicated that *PtrAPX* genes perform important functions in the responses to phytohormones and abiotic stress. For example, *PtrAPX3* and *8* contained six and eight phytohormone- and abiotic stress-related *cis*-elements in the promoter, respectively. Notably, *PtrAPX9* was strongly induced by drought stress in leaves at all time points (Figure 8B), but no drought-related *cis*-element (MBS) was detected in the promoter sequence. Thus, we speculate that *PtrAPX9* may interact with other drought-related genes in the response to drought stress.

Drought and salinity stress have a negative effect on plant development and growth [56,57]. Under exposure to drought and high salinity, plants produce substantially higher quantities of ROS, including H_2_O_2_, in chloroplasts and peroxisomes of photosynthesizing cells [58]. Accumulation of excessive ROS and H_2_O_2_ may cause cellular injury [59]. Ascorbate peroxidase catalyzes the conversion of H_2_O_2_ to H_2_O and O_2_, and scavenges ROS to protect against the toxic effects of ROS accumulation in higher plants [60]. Previous research has shown that *AtAPX1* performs an important function in the drought stress response by protecting chloroplasts from oxidative damage in *Arabidopsis*. *OsAPX2* is induced by salinity stress and transgenic lines of *Arabidopsis* and *Medicago sativa* show enhanced salt tolerance [23,61]. In the present study, expression of most *PtrAPX* genes was induced by drought and salinity stress, which indicated that these genes play an important role in resistance to drought and salinity stress. *PtrAPX6* expression was significantly up-regulated by salinity stress but was down-regulated under drought stress; in contrast, *PtrAPX11* was up-regulated under drought stress but was suppressed under salinity stress. These results indicated that *PtrAPX* genes perform different physiological and biochemical functions in response to environmental stresses. We observed that *PtrAPX10* was significantly upregulated in leaves under drought stress at 3, 6, and 12 h, at which point it was up-regulated approximately 25-fold compared with that of the control (Figure 8B). Interestingly, *PtrAPX10* was strongly suppressed at all time points in roots (Figure 8A,B). This result suggested that *PtrAPX* genes performed different functions in roots and leaves.

Abscisic acid plays an important role in drought stress [62]. Drought stress is known to trigger an increase in ABA content [63]. Moreover, a variety of drought stress-responsive genes are induced by exogenous ABA treatment [64]. Based on the present RT-qPCR results, all *PtrAPX* genes, except *PtrAPX5*, *6*, *7*, and *8*, were induced by both drought stress and ABA treatment. Notably, *PtrAPX9* and *10* were significantly up-regulated in leaves under drought stress and ABA treatment. In maize, the majority of *ZmAPX* genes are induced by ABA treatment, except for *ZmAPX3* and *6*, which are nonresponsive to ABA treatment [25]. The PEG treatment led to similar expression patterns as those observed in response to ABA treatment [25]. The present results are consistent with those reported for maize and suggested that the *PtrAPX* gene family may play an important role in cross-talk between drought stress and ABA treatment. In sugarcane (*Saccharum officinarum*) [65], *APX6* is down-regulated in response to drought and salinity stimuli, but respond positively to ABA treatment. In the present study, *PtrAPX8*, which is homologous to *ScAPX6*, exhibited a similar expression pattern in that expression was suppressed by drought and salinity stress, and was significantly up-regulated by exogenous ABA. The present RT-qPCR data were highly consistent with the aforementioned previous results, thus indicating that homologous genes may perform similar functions in different species.

High salinity, which is considered to be a severe abiotic environmental stress factor, influences woody plant growth and development [66]. Salinity stress may alter biochemical pathways and physiological responses mediated by increased production of ROS [67]. A number of *APX* genes are induced by salt stress [51], such as the cytosolic *OsAPX2* and *7* [11]. An increasing body of evidence has shown that the rapid increase in *APX* mRNA levels may maintain the high activity of APX in the cytosol to protect the cellular components from ROS-induced oxidative damage in response to salinity stress [11]. Moreover, *PtrAPX1* located in chloroplasts and/or mitochondria was stongly induced by high salinity in leaves (Figure 9). A previous study showed that *PtomtAPX* is dual targeted to both chloroplast and mitochondria of *Populus tomentosa* Carr, and showed the same expression pattern under salt stress [30]. Based on the present phylogenetic reconstruction (Figure 1), cytosolic *PtrAPX6* is homologous to *OsAPX2*, and the relative expression level or *PtrAPX6* in roots was increased approximately 16-fold at 3 and 6 h under salt stress. Over-expression of *PpAPX* in transgenic tobacco also showed improved drought resistance and salt tolerance at the vegetative stage [29]. These results suggested that *PtrAPX* genes may play an important role in the salinity stress response.

Excessive NH_4_^+^ concentrations can induce accumulation of ROS in *Arabidopsis* [68] and rice [69]. Plants protect cells from oxidative damage by increasing the expression level of genes encoding antioxidant enzymes [70]. *OsAPX1* and *2* are upregulated under NH_4_Cl treatment, and activate the antioxidant defense mechanism and modulate ROS homeostasis [69]. In the current study, *PtrAPX6*, *9*, and *10* exhibited higher expression levels in roots and leaves compared with those of the control under NH_4_^+^ treatment. This result suggested that these *PtrAPX* genes play a dominant role in the removal of ROS to enhance tolerance to excessive NH_4_^+^ concentrations.

## 5. Conclusions

Eleven *APX* genes that contain the conserved ascorbic acid peroxidase domain were identified in the *P. trichocarpa* genome. The phylogenetic analysis revealed that the PtrAPX proteins were classifiable into three classes. The chromosomal locations, gene duplications, and promoter *cis*-elements of the *PtrAPX* gene family were predicted. A systematic approach was used to examine the roles of the *PtrAPX* genes in the responses to abiotic stress (drought, salinity, and high NH_4_^+^ concentration) and phytohormone treatment (ABA) in relation to promoter *cis*-elements and RT-qPCR analyses. The present findings provide an improved understanding of the evolution and functions of the *APX* gene family in *P. trichocarpa*, and present a framework for further detailed analysis of this gene family.

## Figures and Tables

**Table 1:** Identifiers and physicochemical properties for the 11 *APX* gene family members in *P. trichocarpa*.

Name	Homologous Gene in Poplar	Gene ID	Location	Protein Length (a.a.)	Aliphatic Index	Grand Average of Hydropathicity (GRAVY)
PtrAPX1	PtomtAPX ^a^	Potri.002G081900	Chloroplast/Mitochondrion	377	72.6	−0.491
PtrAPX2	PpAPX ^b^	Potri.004G174500	Cytoplasm	286	84.23	−0.344
PtrAPX3	-	Potri.005G112200	Cytoplasm	287	85.05	−0.336
PtrAPX4	-	Potri.005G161900	Chloroplast	347	81.59	−0.27
PtrAPX5	PtosAPX ^c^	Potri.005G179200	Chloroplast/Mitochondrion	486	82.41	−0.25
PtrAPX6	-	Potri.006G089000	Cytoplasm	96	92.5	0.255
PtrAPX7	-	Potri.006G132200	Cytoplasm	268	89.48	−0.165
PtrAPX8	-	Potri.006G254500	Chloroplast	337	79.91	−0.267
PtrAPX9	-	Potri.009G015400	Cytoplasm	251	80.48	−0.262
PtrAPX10	-	Potri.009G134100	Cytoplasm	286	81.57	−0.322
PtrAPX11	-	Potri.016G084800	Cytoplasm	250	74.96	−0.44

a: identified mitochondria APX gene in *Populus tomentosa* Carr [30]; b: identified *Populus* peroxisomal APX gene in *Populus tomentosa* [28,29] c: identified chloroplastic and/or mitochondrial APX gene in *Populus tomentosa Carr* [30].

**Table 2:** Ka/Ks ratios for duplicate APX genes in *P. trichocarpa*.

Paralogous Pairs	Ks	Ka	Ka/Ks	Divergent Date (Mya)	Duplicate Type
PtrAPX1–PtrAPX5	0.1955	0.047	0.240409207	16.02	Segmental
PtrAPX2–PtrAPX10	0.2350	0.0412	0.175319148	19.26	Segmental
PtrAPX4–PtrAPX8	1.1706	0.9943	0.849393473	95.95	Segmental
PtrAPX6–PtrAPX9	0.0952	0.1162	1.220588235	7.8	Segmental

## Data Availability

The data that support the findings of this study are available from the corresponding author upon reasonable request.

## Supplementary Materials

The following are available online at https://www.mdpi.com/2073-4425/12/3/334/s1, Table S1: Primer pairs for real-time quantitative PCR. Table S2: Statistical analysis of RT-qPCR. Table S3: Motif sequences of *PtrAPX* genes. Table S4: Secondary structure of the *PtrAPX* proteins. Table S5: GO annotation of the *PtrAPX* genes. File 1: The accession numbers and amino acid sequences of *P. trichocarpa*, *Arabidopsis* and rice. File 2: The templates and PDB files of each PtrAPXs.

## References

[1] Mishra P., Bhoomika K., Dubey R.S. (2013). Differential responses of antioxidative defense system to prolonged salinity stress in salt-tolerant and salt-sensitive Indica rice (*Oryza sativa* L.) seedlings. Protoplasma.

[2] Davletova S., Rizhsky L., Liang H., Shengqiang Z., Oliver D.J., Coutu J., Shulaev V., Schlauch K., Mittler R. (2005). Cytosolic ascorbate peroxidase 1 is a central component of the reactive oxygen gene network of Arabidopsis. Plant Cell.

[3] Foyer C.H., Shigeoka S. (2011). Understanding oxidative stress and antioxidant functions to enhance photosynthesis. Plant Physiol..

[4] Maruta T., Sawa Y., Shigeoka S., Ishikawa T. (2016). Diversity and Evolution of Ascorbate Peroxidase Functions in Chloroplasts: More Than Just a Classical Antioxidant Enzyme?. Plant Cell Physiol..

[5] Shigeoka S., Ishikawa T., Tamoi M., Miyagawa Y., Takeda T., Yabuta Y., Yoshimura K. (2002). Regulation and function of ascorbate peroxidase isoenzymes. J. Exp. Bot..

[6] Apel K., Hirt H. (2004). Reactive oxygen species: Metabolism, oxidative stress, and signal transduction. Annu. Rev. Plant Biol..

[7] Panchuk I.I., Volkov R.A., Schoffl F. (2002). Heat stress- and heat shock transcription factor-dependent expression and activity of ascorbate peroxidase in Arabidopsis. Plant Physiol..

[8] Narendra S., Venkataramani S., Shen G., Wang J., Pasapula V., Lin Y., Kornyeyev D., Holaday A.S., Zhang H. (2006). The Arabidopsis ascorbate peroxidase 3 is a peroxisomal membrane-bound antioxidant enzyme and is dispensable for Arabidopsis growth and development. J. Exp. Bot..

[9] Chew O., Whelan J., Millar A.H. (2003). Molecular definition of the ascorbate-glutathione cycle in Arabidopsis mitochondria reveals dual targeting of antioxidant defenses in plants. J. Biol. Chem..

[10] Lazzarotto F., Teixeira F.K., Rosa S.B., Dunand C., Fernandes C.L., Fontenele A.V., Silveira J.A., Verli H., Margis R., Margis-Pinheiro M. (2011). Ascorbate peroxidase-related (APx-R) is a new heme-containing protein functionally associated with ascorbate peroxidase but evolutionarily divergent. New Phytol..

[11] Teixeira F.K., Menezes-Benavente L., Galvao V.C., Margis R., Margis-Pinheiro M. (2006). Rice ascorbate peroxidase gene family encodes functionally diverse isoforms localized in different subcellular compartments. Planta.

[12] Teixeira F.K., Menezes-Benavente L., Margis R., Margis-Pinheiro M. (2004). Analysis of the molecular evolutionary history of the ascorbate peroxidase gene family: Inferences from the rice genome. J. Mol. Evol..

[13] Caverzan A., Bonifacio A., Carvalho F.E., Andrade C.M., Passaia G., Schunemann M., Maraschin F.S., Martins M.O., Teixeira F.K., Rauber R. (2014). The knockdown of chloroplastic ascorbate peroxidases reveals its regulatory role in the photosynthesis and protection under photo-oxidative stress in rice. Plant Sci..

[14] Wu B., Li L., Qiu T., Zhang X., Cui S. (2018). Cytosolic APX2 is a pleiotropic protein involved in H2O2 homeostasis, chloroplast protection, plant architecture and fertility maintenance. Plant Cell Rep..

[15] Panchuk I.I., Zentgraf U., Volkov R.A. (2005). Expression of the Apx gene family during leaf senescence of *Arabidopsis thaliana*. Planta.

[16] Khanna-Chopra R., Jajoo A., Semwal V.K. (2011). Chloroplasts and mitochondria have multiple heat tolerant isozymes of SOD and APX in leaf and inflorescence in *Chenopodium album*. Biochem. Biophys. Res. Commun..

[17] Pnueli L., Liang H., Rozenberg M., Mittler R. (2003). Growth suppression, altered stomatal responses, and augmented induction of heat shock proteins in cytosolic ascorbate peroxidase (Apx1)-deficient Arabidopsis plants. Plant J..

[18] Koussevitzky S., Suzuki N., Huntington S., Armijo L., Sha W., Cortes D., Shulaev V., Mittler R. (2008). Ascorbate peroxidase 1 plays a key role in the response of *Arabidopsis thaliana* to stress combination. J. Biol. Chem..

[19] Miller G., Suzuki N., Rizhsky L., Hegie A., Koussevitzky S., Mittler R. (2007). Double mutants deficient in cytosolic and thylakoid ascorbate peroxidase reveal a complex mode of interaction between reactive oxygen species, plant development, and response to abiotic stresses. Plant Physiol..

[20] Chen C., Letnik I., Hacham Y., Dobrev P., Ben-Daniel B.H., Vankova R., Amir R., Miller G. (2014). ASCORBATE PEROXIDASE6 protects Arabidopsis desiccating and germinating seeds from stress and mediates cross talk between reactive oxygen species, abscisic acid, and auxin. Plant Physiol..

[21] Ribeiro C.W., Korbes A.P., Garighan J.A., Jardim-Messeder D., Carvalho F., Sousa R., Caverzan A., Teixeira F.K., Silveira J., Margis-Pinheiro M. (2017). Rice peroxisomal ascorbate peroxidase knockdown affects ROS signaling and triggers early leaf senescence. Plant Sci..

[22] Zhang Z., Zhang Q., Wu J., Zheng X., Zheng S., Sun X., Qiu Q., Lu T. (2013). Gene knockout study reveals that cytosolic ascorbate peroxidase 2(OsAPX2) plays a critical role in growth and reproduction in rice under drought, salt and cold stresses. PLoS ONE.

[23] Lu Z., Liu D., Liu S. (2007). Two rice cytosolic ascorbate peroxidases differentially improve salt tolerance in transgenic Arabidopsis. Plant Cell Rep..

[24] Singh N., Mishra A., Jha B. (2014). Over-expression of the peroxisomal ascorbate peroxidase (SbpAPX) gene cloned from halophyte *Salicornia brachiata* confers salt and drought stress tolerance in transgenic tobacco. Mar. Biotechnol..

[25] Liu Y., Yuan Y., Liu Y., Liu Y., Fu J., Zheng J., Wang G. (2012). Gene families of maize glutathione–ascorbate redox cycle respond differently to abiotic stresses. J. Plant Physiol..

[26] Rennenberg H., Wildhagen H., Ehlting B. (2010). Nitrogen nutrition of poplar trees. Plant. Biol..

[27] Tuskan G.A., Difazio S., Jansson S., Bohlmann J., Grigoriev I., Hellsten U., Putnam N., Ralph S., Rombauts S., Salamov A. (2006). The genome of black cottonwood, *Populus trichocarpa* (Torr. & Gray). Science.

[28] Lu H., Han R.L., Jiang X.N. (2009). Heterologous expression and characterization of a proxidomal ascorbate peroxidase from *Populus tomentosa*. Mol. Biol. Rep..

[29] Li Y., Hai R., Du X., Jiang X., Lu H. (2009). Over-expression of a Populus peroxisomal ascorbate peroxidase (PpAPX) gene in tobacco plants enhances stress tolerance. Plant Breed..

[30] Yin B., Zhang J., Liu Y., Pan X., Zhao Z., Li H., Zhang C., Li C., Du X., Li Y. (2019). PtomtAPX, a mitochondrial ascorbate peroxidase, plays an important role in maintaining the redox balance of *Populus tomentosa* Carr. Sci. Rep..

[31] Librado P., Rozas J. (2009). DnaSP v5: A software for comprehensive analysis of DNA polymorphism data. Bioinformatics.

[32] Wei M., Xu X., Li C. (2017). Identification and expression of CAMTA genes in *Populus trichocarpa* under biotic and abiotic stress. Sci. Rep..

[33] Thompson J.D., Gibson T.J., Plewniak F., Jeanmougin F., Higgins D.G. (1997). The CLUSTAL_X windows interface: Flexible strategies for multiple sequence alignment aided by quality analysis tools. Nucleic Acids Res..

[34] Kumar S., Stecher G., Tamura K. (2016). MEGA7: Molecular Evolutionary Genetics Analysis Version 7.0 for Bigger Datasets. Mol. Biol. Evol..

[35] Sjodin A., Street N.R., Sandberg G., Gustafsson P., Jansson S. (2009). The Populus Genome Integrative Explorer (PopGenIE): A new resource for exploring the Populus genome. New Phytol..

[36] Livak K.J., Schmittgen T.D. (2001). Analysis of relative gene expression data using real-time quantitative PCR and the 2(-Delta Delta C(T)) Method. Methods.

[37] Schmittgen T.D., Livak K.J. (2008). Analyzing real-time PCR data by the comparative C(T) method. Nat. Protoc..

[38] Pettengill E.A., Parmentier-Line C., Coleman G.D. (2012). Evaluation of qPCR reference genes in two genotypes of Populus for use in photoperiod and low-temperature studies. BMC Res. Notes.

[39] Pettengill E.A., Pettengill J.B., Coleman G.D. (2013). Elucidating the evolutionary history and expression patterns of nucleoside phosphorylase paralogs (vegetative storage proteins) in Populus and the plant kingdom. BMC Plant Biol..

[40] Liao G.L., Liu Q., Li Y.Q., Zhong M., Huang C.H., Jia D.F., Xu X.B. (2020). Identification and expression profiling analysis of ascorbate peroxidase gene family in *Actinidia chinensis* (Hongyang). J. Plant Res..

[41] Kwong S., Woods A.E., Mirtschin P.J., Ge R., Kini R.M. (2009). The recruitment of blood coagulation factor X into snake venom gland as a toxin: The role of promoter cis-elements in its expression. Thromb. Haemost..

[42] Redfern O.C., Dessailly B.H., Dallman T.J., Sillitoe I., Orengo C.A. (2009). FLORA: A novel method to predict protein function from structure in diverse superfamilies. PLoS Comput. Biol..

[43] Xu Z., Gao L., Tang M., Qu C., Huang J., Wang Q., Yang C., Liu G., Yang C. (2017). Genome-wide identification and expression profile analysis of CCH gene family in Populus. Peerj.

[44] Yang X., Kalluri U.C., Jawdy S., Gunter L.E., Yin T., Tschaplinski T.J., Weston D.J., Ranjan P., Tuskan G.A. (2008). The F-box gene family is expanded in herbaceous annual plants relative to woody perennial plants. Plant Physiol..

[45] Zhuang Y., Wang C., Zhang Y., Chen S., Wang D., Liu Q., Zhou G., Chai G. (2019). Overexpression of PdC3H17 Confers Tolerance to Drought Stress Depending on Its CCCH Domain in Populus. Front. Plant Sci..

[46] Li J., Sun P., Xia Y., Zheng G., Sun J., Jia H. (2019). A Stress-Associated Protein, PtSAP13, from *Populus trichocarpa* Provides Tolerance to Salt Stress. Int. J. Mol. Sci..

[47] Liu R., Wu M., Liu H.L., Gao Y.M., Chen J., Yan H.W., Xiang Y. (2020). Genome-wide identification and expression analysis of the NF-Y transcription factor family in Populus. Physiol. Plant..

[48] Storozhenko S., De Pauw P., Van Montagu M., Inze D., Kushnir S. (1998). The heat-shock element is a functional component of the Arabidopsis APX1 gene promoter. Plant Physiol..

[49] Santos M., Gousseau H., Lister C., Foyer C., Creissen G., Mullineaux P. (1996). Cytosolic ascorbate peroxidase from *Arabidopsis thaliana* L. is encoded by a small multigene family. Planta.

[50] Wu B., Wang B. (2019). Comparative analysis of ascorbate peroxidases (APXs) from selected plants with a special focus on *Oryza sativa* employing public databases. PLoS ONE.

[51] Najami N., Janda T., Barriah W., Kayam G., Tal M., Guy M., Volokita M. (2008). Ascorbate peroxidase gene family in tomato: Its identification and characterization. Mol. Genet. Genomics.

[52] Tao C., Jin X., Zhu L., Xie Q., Wang X., Li H. (2018). Genome-wide investigation and expression profiling of APX gene family in *Gossypium hirsutum* provide new insights in redox homeostasis maintenance during different fiber development stages. Mol. Genet. Genomics.

[53] Ozyigit I.I., Filiz E., Vatansever R., Kurtoglu K.Y., Koc I., Ozturk M.X., Anjum N.A. (2016). Identification and Comparative Analysis of H2O2-Scavenging Enzymes (*Ascorbate peroxidase* and *Glutathione peroxidase*) in Selected Plants Employing Bioinformatics Approaches. Front. Plant Sci..

[54] Xu G., Guo C., Shan H., Kong H. (2012). Divergence of duplicate genes in exon-intron structure. Proc. Natl. Acad. Sci. USA.

[55] Yang Y., Ahammed G.J., Wan C., Liu H., Chen R., Zhou Y. (2019). Comprehensive Analysis of TIFY Transcription Factors and Their Expression Profiles under Jasmonic Acid and Abiotic Stresses in Watermelon. Int. J. Genomics.

[56] Takahashi F., Kuromori T., Sato H., Shinozaki K. (2018). Regulatory Gene Networks in Drought Stress Responses and Resistance in Plants. Adv. Exp. Med. Biol..

[57] Chen S., Polle A. (2010). Salinity tolerance of *Populus*. Plant Biol..

[58] Del R.L., Sandalio L.M., Corpas F.J., Palma J.M., Barroso J.B. (2006). Reactive oxygen species and reactive nitrogen species in peroxisomes. Production, scavenging, and role in cell signaling. Plant Physiol..

[59] Dat J., Vandenabeele S., Vranova E., Van Montagu M., Inze D., Van Breusegem F. (2000). Dual action of the active oxygen species during plant stress responses. Cell Mol. Life Sci..

[60] Chen Y., Cai J., Yang F.X., Zhou B., Zhou L.R. (2015). Ascorbate peroxidase from *Jatropha curcas* enhances salt tolerance in transgenic Arabidopsis. Genet. Mol. Res..

[61] Guan Q., Takano T., Liu S. (2012). Genetic transformation and analysis of rice OsAPx2 gene in *Medicago sativa*. PLoS ONE.

[62] Fujita Y., Fujita M., Satoh R., Maruyama K., Parvez M.M., Seki M., Hiratsu K., Ohme-Takagi M., Shinozaki K., Yamaguchi-Shinozaki K. (2005). AREB1 is a transcription activator of novel ABRE-dependent ABA signaling that enhances drought stress tolerance in Arabidopsis. Plant Cell.

[63] Gao Y.F., Liu J.K., Yang F.M., Zhang G.Y., Wang D., Zhang L., Ou Y.B., Yao Y.A. (2020). The WRKY transcription factor WRKY8 promotes resistance to pathogen infection and mediates drought and salt stress tolerance in *Solanum lycopersicum*. Physiol. Plant..

[64] Yamaguchi-Shinozaki K., Shinozaki K. (2006). Transcriptional regulatory networks in cellular responses and tolerance to dehydration and cold stresses. Annu. Rev. Plant Biol..

[65] Liu F., Huang N., Wang L., Ling H., Sun T., Ahmad W., Muhammad K., Guo J., Xu L., Gao S. (2017). A Novel L-ascorbate Peroxidase 6 Gene, ScAPX6, Plays an Important Role in the Regulation of Response to Biotic and Abiotic Stresses in Sugarcane. Front. Plant Sci..

[66] Chen N., Tong S., Tang H., Zhang Z., Liu B., Lou S., Liu J., Liu H., Ma T., Jiang Y. (2020). The PalERF109 transcription factor positively regulates salt tolerance via PalHKT1;2 in *Populus alba* var. pyramidalis. Tree Physiol..

[67] Zhu C., Sanahuja G., Yuan D., Farre G., Arjo G., Berman J., Zorrilla-Lopez U., Banakar R., Bai C., Perez-Massot E. (2013). Biofortification of plants with altered antioxidant content and composition: Genetic engineering strategies. Plant. Biotechnol. J..

[68] Patterson K., Cakmak T., Cooper A., Lager I., Rasmusson A.G., Escobar M.A. (2010). Distinct signalling pathways and transcriptome response signatures differentiate ammonium- and nitrate-supplied plants. Plant Cell Environ..

[69] Xie Y., Mao Y., Xu S., Zhou H., Duan X., Cui W., Zhang J., Xu G. (2015). Heme-heme oxygenase 1 system is involved in ammonium tolerance by regulating antioxidant defence in *Oryza sativa*. Plant Cell Environ..

[70] Yang S., Hao D., Jin M., Li Y., Liu Z., Huang Y., Chen T., Su Y. (2020). Internal ammonium excess induces ROS-mediated reactions and causes carbon scarcity in rice. BMC Plant Biol..

