# Supplementary material for: Identification and Characterization of the APX Gene Family and Its Expression Pattern under Phytohormone Treatment and Abiotic Stress in Populus trichocarpa
Source: Genes (Basel). 2021 Feb 25;12(3):334. doi: 10.3390/genes12030334 (PMC7996185; doi:10.3390/genes12030334)
Supplement: Supplementary file 1 [file genes-12-00334-s001.zip › Supplementary file 1.docx]

>OsAPX1 (LOC_Os03g17690)

MAKNYPVVSAEYQEAVEKARQKLRALIAEKSCAPLMLRLAWHSAGTFDVSSKTGGPFGTMKTPAELSHAANAGLDIAVRMLEPIKEEIPTISYADFYQLAGVVAVEVSGGPAVPFHPGREDKPAPPPEGRLPDATKGSDHLRQVFGAQMGLSDQDIVALSGGHTLGRCHKERSGFEGPWTRNPLQFDNSYFTELLSGDKEGLLQLPSDKALLSDPAFRPLVEKYAADEKAFFEDYKEAHLKLSELGFADA*

>OsAPX2 (LOC_Os07g49400)

MGSKSYPTVSDEYLAAVGKAKRKLRGLIAEKNCAPLMLRLAWHSAGTFDVSSRTGGPFGTMKNPGEQSHAANAGLDIAVRLLDPIKDQLPILSYADFYQLAGVVAVEVTGGPEVPFHPGRQDKPEPPPEGRLPDATQGSDHLRQVFSAQMGLSDKDIVALSGGHTLGRCHKERSGFEGAWTSNPLIFDNSYFTELVSGEKEGLLQLPSDKALMADPAFRPLVEKYAADEDAFFADYAEAHLKLSELGFAEE*

>OsAPX3 (LOC_Os04g14680)

MSAAPVVDAEYMAEVERARRDLRALIASKSCAPIMLRLAWHDAGTYDKATKTGGPNGSIRFPQEYSHAANAGIKIAIDLLEPMKQKHPKITYADLYQLAGVVAVEVTGGPTIDYVPGRRDSSDSPEEGRLPDAKKGAAHLREVFYRMGLSDKDIVALSGGHTLGKARPERSGFDGAWTKDPLKFDNSYFIELLKENSEGLLKLPTDKALVEDPTFRRYVELYAKDEDAFFRDYAESHKKLSELGFTPPRSAFIYKSCQKPKSLLMQTAAGVAVAAAVVAWAYLCESNKRLG*

>OsAPX4 (LOC_Os08g43560)

MAAPVVDAEYLRQVDRARRHLRALISSKGCAPIMLRLAWHDAGTYDVNTKTGGANGSIRYEEEYTHGSNAGLKIAIDLLEPIKAKSPKITYADLYQLAGVVAVEVTGGPTVEFIPGRRDSSVCPREGRLPDAKKGALHLRDIFYRMGLSDKDIVALSGGHTLGRAHPERSGFEGAWTQEPLKFDNSYFLELLKGESEGLLKLPTDKALLEDPSFRRYVDLYARDEDTFFKDYAESHKKLSELGFTPRSSGPASTKSDLSTGAVLAQSAVGVAVAAAVVIVSYLYEASKKSK*

>OsAPX5 (LOC_Os12g07830)

MAVVHRILRRGLSAASPLPSLRGLLLVSPQELGRRPASSSSSAAAAAGDVEAELRAAREDVRQLLKSNPCHPILVRLGWHDAGTYDKNITEWPKCGGANGSLRFGVELVHAANKGLLKALFLVIPIKSKYAGVTYADIFQLASATAIEEAGGPKIPMIYGRADVADGEECPPEGRLPAADPPSPAEHLREVFYRMGLSDKEIVALSGAHTLGRARPERSGWGKPETKYTENGPGAPGGQSWTSEWLKFDNSYFKEIKERRDEDLLVLPTDAVLFEDSSFKIHAEKYAEDQDAFFEDYAEAHAKLSNLGAKFDPPKGISLE*

>OsAPX6 (LOC_Os12g07820)

MAVVHRLLRRGLSAASPLPSLQELGRRPASSSAAAAGDAAAELRGAREDVKQLLKSTSCHPILVRLGWHDAGTYDKNITEWPKCGGANGSLRFEIELKHAANAGLVNALKLIQPIKDKHAGVTYADLFQLASATAIEEAGGPKIPMIYGRVDVAAPEQCPPEGRLPAAGPPSPAEHLREVFYRMGLSDKEIVALSGAHTLGRSRPERSGWGKPETKYTKNGPGAPGGQSWTSQWLKFDNSYFKDIKERRDEDLLVLPTDAVLFEDSSFKIYAEKYAADQDAFFEDYAEAHAKLSNLGAKFDPPKGISLE*

>OsAPX7 (LOC_Os04g35520)

MAAQRLAALHAAAPSAFSSTSSASHGRPAARSSTTALLPVALPRASATLRAAPSRLLPQEAKAAGSGRSVMCMASASASAASAAVASGAAELKAAREDIRELLKTTHCHPILVRLGWHDSGTYDKNIKEWPQRGGANGSLRFDVELKHGANAGLVNALKLVQPIKDKYPNISYADLFQLASATAIEEAGGPKIPMTYGRIDVTGPEQCPPEGKLPDAGPSAPADHLRKVFYRMGLDDKEIVVLSGAHTLGRSRPERSGWGKPETKYTKNGPGAPGGQSWTAEWLKFDNSYFKEIKEKRDQDLLVLPTDAALFEDPTFKVYAEKYAEDQEAFFKDYAGAHAKLSNLGAKFNPPEGFTLDG*

>OsAPX8 (LOC_Os02g34810)

MAERIAASLLPAASPSPAPSPPPPRPRVSAAAAASFPCCSTSAGGLRLRSRPSRFPQKAATTRSGRAGAGARAVVRCMAAAAVAASDAAQLKSAREDIREILKTTYCHPIMVRLGWHDSGTYDKNIEEWPQRGGADGSLRFDAELSHGANAGLINALKLIQPIKDKYPGITYADLFQLASATAIEEAGGPKIPMKYGRVDVTAAEQCPPEGRLPDAGPRVPADHLREVFYRMGLDDKEIVALSGAHTLGRSRPDRSGWGKPETKYTKDGPGEPGGQSWTVEWLKFDNSYFKDIKEQRDQDLLVLPTDAALFEDPSFKVYAEKYAEDQEAFFKDYAEAHAKLSDLGAKFDPPEGFSLDDEPAVEEKDPEPAPAPAAAPPPPPVEEKKEAEPTPVPVTVGAAVASSPADDNNGAAPQPEPFVAAKYSYGKKELSDSMKQKIRAEYEGFGGSPDKPLQSNYFLNIMLLIGGLAFLTSLLGS*

>AtAPX1 (At1G07890)

MTKNYPTVSEDYKKAVEKCRRKLRGLIAEKNCAPIMVRLAWHSAGTFDCQSRTGGPFGTMRFDAEQAHGANSGIHIALRLLDPIREQFPTISFADFHQLAGVVAVEVTGGPDIPFHPGREDKPQPPPEGRLPDATKGCDHLRDVFAKQMGLSDKDIVALSGAHTLGRCHKDRSGFEGAWTSNPLIFDNSYFKELLSGEKEGLLQLVSDKALLDDPVFRPLVEKYAADEDAFFADYAEAHMKLSELGFADA*

>AtAPX2 (AT3G09640)

MVKKSYPEVKEEYKKAVQRCKRKLRGLIAEKHCAPIVLRLAWHSAGTFDVKTKTGGPFGTIRHPQELAHDANNGLDIAVRLLDPIKELFPILSYADFYQLAGVVAVEITGGPEIPFHPGRLDKVEPPPEGRLPQATKGVDHLRDVFGRMGLNDKDIVALSGGHTLGRCHKERSGFEGAWTPNPLIFDNSYFKEILSGEKEGLLQLPTDKALLDDPLFLPFVEKYAADEDAFFEDYTEAHLKLSELGFADKE*

>AtAPX3 (AT4G35000)

MAAPIVDAEYLKEITKARRELRSLIANKNCAPIMLRLAWHDAGTYDAQSKTGGPNGSIRNEEEHTHGANSGLKIALDLCEGVKAKHPKITYADLYQLAGVVAVEVTGGPDIVFVPGRKDSNVCPKEGRLPDAKQGFQHLRDVFYRMGLSDKDIVALSGGHTLGRAHPERSGFDGPWTQEPLKFDNSYFVELLKGESEGLLKLPTDKTLLEDPEFRRLVELYAKDEDAFFRDYAESHKKLSELGFNPNSSAGKAVADSTILAQSAFGVAVAAAVVAFGYFYEIRKRMK*

>AtAPX4 (AT4G09010)

MGGVSFLSTVPSFTNTTNHQHLTTLSSSSHRSAVIRCSKIEPQVSGESLAFHRRDVLKLAGTAVGMELIGNGFINNVGDAKAADLNQRRQRSEFQSKIKILLSTTIKAKPELVPSLLKLALNDAMTYDKATKSGGANGSIRFSSELSRAENEGLSDGLSLIEEVKKEIDSISKGGPISYADIIQLAGQSAVKFTYLASAIRKCGGNEEKGNLLYTAYGSAGQWGLFDRNFGRSDATEADPEGRVPQWGKATVQEMKDKFIAVGLGPRQLAVMSAFLGPDQAATEQLLATDPQVAPWVQKYQRSRETVSQTDYEVDLITAFTKLSCLGQQINFEAYTYPVERINLSKLKL*

>AtAPX5 (AT4G35970)

MAVNVDAEYLKEIEKTRRDLRALISSRNCAPIMLRLAWHDAGTYDAKKKTGGANGSIRFKEELNRPHNKGLEKAVAFCEEVKAKHPRVSYADLYQLAGVVAVEVTGGPAIPFTPGRKDADSADDGELPNPNEGASHLRTLFSRMGLLDRDIVALSGGHTLGRAHKERSDFEGPWTQDPLKFDNSYFVELLKGETPGLLQLKTDKALLDDPKFHPFVKLYAKDEDMFFKAYAISHKKLSELGFNPPRRIPSAVTQQTLGIAVAAAVVIFTICYEASRRGK*

>AtAPX6 (AT4G32320)

MTTTTASLVKTFLFRCDSFSSFKFKCKFESPAKTRLLSPATEKHVVRSSRAWRIRCLSDDPGSSHVFVASRRKMVVLLSTVQLLSHMLPQNGNAAEIYPVMQNEIRKVVTKGKAAGVLRLVFHDAGTFELDDHSGGINGSIAYELERPENIGLKKSLKVLAKAKVKVDEIQPVSWADMISVAGSEAVSICGGPTIPVVLGRLDSAQPDPEGKLPPETLSASGLKECFKRKGFSTQELVALSGAHTIGSKGFGDPTVFDNAYYKILLEKPWTSTSKMTSMVGLPSDHALVQDDECLRWVKRYAEDQDKFFEDFTNAYIKLVNSGAKWNML*

>AtsAPX (AT4G08390)

MAERVSLTLNGTLLSPPPTTTTTTMSSSLRSTTAASLLLRSSSSSSRSTLTLSASSSLSFVRSLVSSPRLSSSSSLSQKKYRIASVNRSFNSTTAATKSSSSDPDQLKNAREDIKELLSTKFCHPILVRLGWHDAGTYNKNIKEWPQRGGANGSLRFDIELKHAANAGLVNALNLIKDIKEKYSGISYADLFQLASATAIEEAGGPKIPMKYGRVDASGPEDCPEEGRLPDAGPPSPATHLREVFYRMGLDDKDIVALSGAHTLGRSRPERSGWGKPETKYTKEGPGAPGGQSWTPEWLKFDNSYFKEIKEKRDEDLLVLPTDAAIFEDSSFKVYAEKYAADQDAFFKDYAVAHAKLSNLGAEFNPPEGIVI*

>AttAPX (AT1G77490)

MSVSLSAASHLLCSSTRVSLSPAVTSSSSSPVVALSSSTSPHSLGSVASSSLFPHSSFVLQKKHPINGTSTRMISPKCAASDAAQLISAKEDIKVLLRTKFCHPILVRLGWHDAGTYNKNIEEWPLRGGANGSLRFEAELKHAANAGLLNALKLIQPLKDKYPNISYADLFQLASATAIEEAGGPDIPMKYGRVDVVAPEQCPEEGRLPDAGPPSPADHLRDVFYRMGLDDKEIVALSGAHTLGRARPDRSGWGKPETKYTKTGPGEAGGQSWTVKWLKFDNSYFKDIKEKRDDDLLVLPTDAALFEDPSFKNYAEKYAEDVAAFFKDYAEAHAKLSNLGAKFDPPEGIVIENVPEKFVAAKYSTGKKELSDSMKKKIRAEYEAIGGSPDKPLPTNYFLNIIIAIGVLVLLSTLFGGNNNSDFSGF*

>PtrAPX1 (POPTR_0002s08240)

MAQYPSSLTLSPPKSQPMASLRGSAATVRLLHSASRVRLSLSSASSSLSISSSSSYSPSSLKCLQFSPLAPHIFKDQNRSSMSTVAAASDPAQLKSAREDIKELLKSKSCHPILVRLGWHDSGTYNKNIEEWPRMGGANGSLRFDIELKHAANAGLVNALKLIKPIKDKYSGVTYADLFQLASATAIEEAGGPKIPMKYGRVDVSAPEECPEEGRLPAAGPPKPADHLREVFYRMGLNDKEIVALSGAHTLGRSRPERSGWGKPETKYTKNGPGAPGGQSWTAEWLKFDNSYFKDIKQRKDDDLLVLPTDAALFEDPSFKVYAEKYAEDQEAFFKDYAEAHAKLSNLGAKFDPQEGIVLDGVAGEKFMAAKYSSGKD*

>PtrAPX2 (POPTR_0004s18030)

MALPVVDTEYLKEIEKARRDLRAVIAYKNCAPIMLRLAWHDAGTYDKNTKTGGANGSIRNEEECSHGSNNGLKIAIDSCEEVKVKHPKITYADLYQLAGVVAVEVTGGPTIDFVPGRRDSNTCPKEGRLPNAKLGSPHLRDIFYRMGLSDKDIVALSGGHTLGRAHPERSGFDGPWTQEPLKFDNSYFVELLKGQTEGLLKLPTDTALLDDPDFRPYVELYAKDEEAFFRDYAASHKKLSELGFTPRSSVVKVKDSTVLAQSAVGVAVAAAVVILGYFYEVRKKMN*

>PtrAPX3 (POPTR_0005s11430)

MAGKVVDAEYSKEIEKARRDLRALIASKSCAPIMLRLAWHDAGTYDAKTKTGGPDGSIRNEKELAHAANNGIKIAIDFCEGIKAKHPKITYADLYQLAGVVAVEVTGGPTIDFVPGRKDSPESPEEGRLPDAKQGATHLRDVFYRMGLSDKDIVALSGGHTLGRAHRDRSGFDGPWTKEPLKFDNSYFQELLKGDSEGLLKLQTDRVLVEDPKFCKYVLLYAEDEDAFFSDYAASHKKLSELGFTPPSSSLKAITKNRTLLAQSAVGVAVAATVIILSYFYEINRRV*

>PtrAPX4 (POPTR_0005s17350)

MGASFLSTLPSLVSVPSLTISTTPTIRCPSHAVAIRCHKMDTDVPGENVLRRREVLKCFGAAVGMELLASSGSFVEMASAADLIQRRQRSEFQSSIRQTLFAAIKGKPELVPYILTLALNDAITYDKATKSGGPNGSIRFSSEISRPENKGLSAALNLIEEAKEEIDSYSKGGPISFADLIPYAGQSAVKATFLASAIRKCGGNEQKGSLLYSAYGSSGQWGLFDRQFGRTDSQEPDPEGRVPQWEKATVQEMKDKFSSIGFGPRQLAVMSAFLGPDQAATEALLATDPDVTPWVQKYQRSRETVSQTDYEVDLITTLTKLSSMGQQINYEAYTYPVRKIELSKLKL*

>PtrAPX5 (POPTR_0005s20140)

MTQRNPILLRNSLYKCACAHHIHFIQNTRRAIQPTDKTGAKSQMAQYLSQLTLSPPKSQPMASLSGGAATTSRLLPSASRVRLSICSASSSLSLASSSSYSPSSLKCLRFSPLIFKDQKRSSMSTVAAASDAAQLKSAREDIKELLKSKFCHPILVRLGWHDAGTYNKNIEEWPKRGGANGSLRFEIELKHAANAGLVDALKLIQPIKDKYSGVTYADLFQMASAAAIEEAGGPKIPMKYGRVDVSVPDECPEEGRLPDAGPPKPADHLREVFYRMGLDDKEIAALSGAHTLGRSRPERSGWGKPETKYTKNGPGAPGGQSWTAEWLKFDNSYFKDIKERKDEDLLVLPTDAALFEDPSFKVYAEKYAEDKEAFFKDYAEAHAKLSNLGAKFDPPEGIMLDGVAGEKFVAAKYSSGKVVITIKLLQNVLLIINHKSSLVFRVAWMIYMAEVYVCKMFIFSLFMIHCRTKKQASCFLVPFLLIIQTG*

>PtrAPX6 (POPTR_0006s08980)

MNCAHLSLCLAWYSAGTFGVKTKTDGPFGTMRYSAELAHGANNGLDIAVRLLEPIKEQFPILSYADFYQLAGVVSVAITGGPEVPFHPGSEPSIVL*

>PtrAPX7 (POPTR_0006s13440)

MGKSYPTVSEEYQKAVEKCKRKLRGLIAEKHCAPLMLRLAWHSAGTFDVHTKTGGPFGTIRHPDELAHGANNGLDIAIRLLEPIKEQFPILSYADFYQLAGVVAVEVTGGPEIPFHPGRPDKSDPPPEGRLPDATKGSDHLRDVFGHMGLSDTDIVALSGGHTLVCSQTWNYVKNFLLILFCLCTIGVCTYIYRGGAIRSVLDSRDPGPPTHLFSTTPISRNSSVERKKVLSSFHQTKLFWRIQSSVPLLKTMLKMRMHSLQIIQKLI*

>PtrAPX8 (POPTR_0006s27060)

(MASLTILGTTKPSSLLSSTLSSTSPQEFKFPAKSQRPFSSTVKFRAKPFKACAFPSNNNDNGEKDDCFSTRRSLLVCISTLPFLFGLHEFLEDLSAKALQSDTNTYMLIKEEVRKVVSKGKAAGVLRLVFHDAGTFEMDGNSGGMNGSIVYELERPENAGLKKSLKILDKAKGEVDAIQQVSWADMIAVAGAEAVSVCGGPTIPVQLGRLDSLEPDAEGKLPRESLDAPGLKQNFKRKGLSTQELVALSGAHTLGSKGFGSPFVFDNSYYKILLEKPWKSSGGMSSMIGLPSDHALVEDDECLRWIKKYADNQNMFFDDFKNAYIKLVNSGARWKSL*)

>PtrAPX9 (POPTR_0009s02070)

MTKNYPTVSEEYSKAVEKAKKKLRSLIAEKSCAPLMLRLAWHSAGTFDVKTKTGGPFGTMRYSAELAHGANNGLDIAVRLLESIKEQFPILSYADFYQLAGVVGVEITGGPEVPFHPGREDKPEPPPEGRLPDATKGSDHLRDVFGHMGLSDKDIVALSGGHTLGRCHKERSGFEGPWTANPLIFDNSYFKELLSGEKEGLLQLPSDKALLSDPIFRPYVDKYAAVCTKNIICCASGACQIHEKLLVSCCC*

>PtrAPX10 (POPTR_0009s13650)

MALPVVDTEYLKEIDKARRDLRALIAYKSCAPLMLRLAWHDAGTYDKNSKTGGANGSIRNEEEYSHGSNSGLKIAIDFCEGVKAKHPKITYADLYQLAGVVAVEVTGGPTIDFVPGRRDSNICPKEGRLPNAKLGSPHLRDIFYRMGLSDKDIVALSGGHTLGRAHPDRSGFEGPWTQEPLKFDNSYFVEMLKGETDGLLKLPTDTALLDDPAFRPYVELYAKDEEAFFRDYAASHKKLSELGFTQGSSGFKVKDTTVLAQSAAGVAVAAAVVILSYFYEVRKKMK*

>PtrAPX11 (POPTR_0016s08580)

MGKCYPTVSEEYQKAVEKCKRKLRGLIAEKHCAPLMLRLAWHSAGTFDVNTKTGGPFGTIRHPDELAHGANNGLDIAVRLLEPLKEQFPNLSYADFYQLAGVVAVEITGGPEVPFHPGRPDKSDPPPEGRLPDATKGSDHLRDVFGHMGLSDKDIVALSGGHTLGRCHKERSGFEGPWTPNPLVFDNSYFKELLSGEKEGLIQLPTDKTLLEDPVFRPLVEKYAADEDAFFADYAEAHMKLSELGFAEAY*
