# Supplementary figures and images for: Identification and Characterization of the APX Gene Family and Its Expression Pattern under Phytohormone Treatment and Abiotic Stress in Populus trichocarpa
Source: Genes (Basel). 2021 Feb 25;12(3):334. doi: 10.3390/genes12030334 (PMC7996185; doi:10.3390/genes12030334)

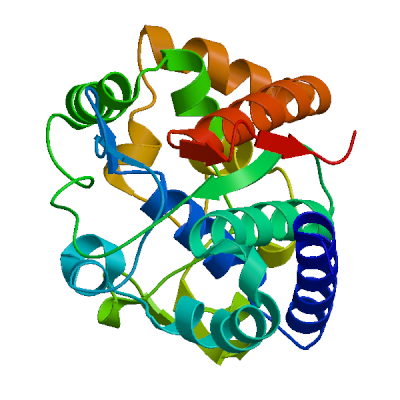

Supplement: Supplementary file 1 [file genes-12-00334-s001.zip › Supplementary file 2/Supplementary file 2/APX1/model/01/01.png]

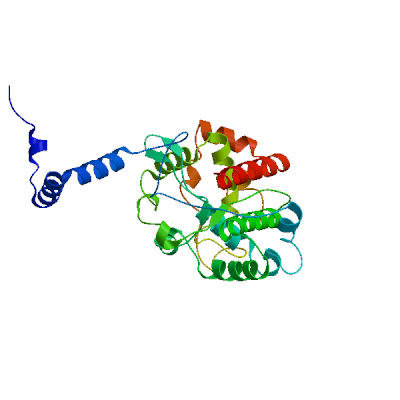

Supplement: Supplementary file 1 [file genes-12-00334-s001.zip › Supplementary file 2/Supplementary file 2/APX1/model/02/02.png]

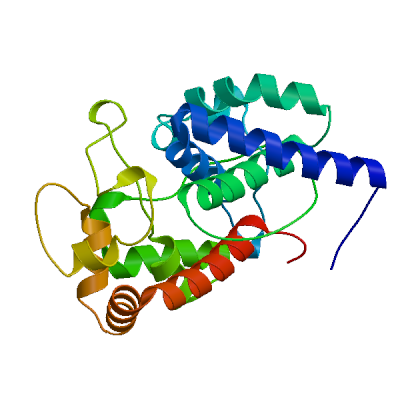

Supplement: Supplementary file 1 [file genes-12-00334-s001.zip › Supplementary file 2/Supplementary file 2/APX10/model/01/01.png]

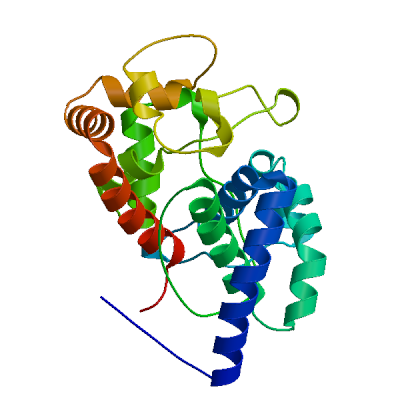

Supplement: Supplementary file 1 [file genes-12-00334-s001.zip › Supplementary file 2/Supplementary file 2/APX11/model/01/01.png]

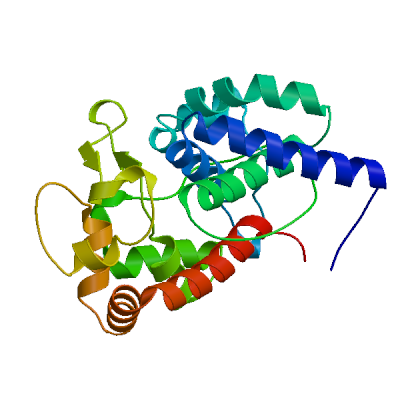

Supplement: Supplementary file 1 [file genes-12-00334-s001.zip › Supplementary file 2/Supplementary file 2/APX2/model/01/01.png]

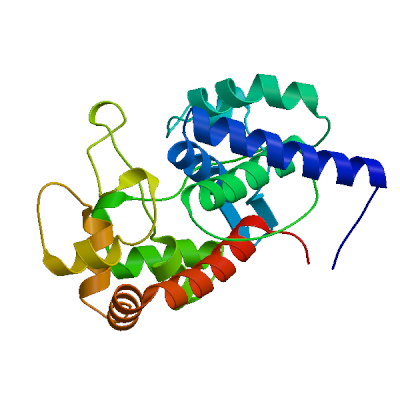

Supplement: Supplementary file 1 [file genes-12-00334-s001.zip › Supplementary file 2/Supplementary file 2/APX3/model/01/01.png]

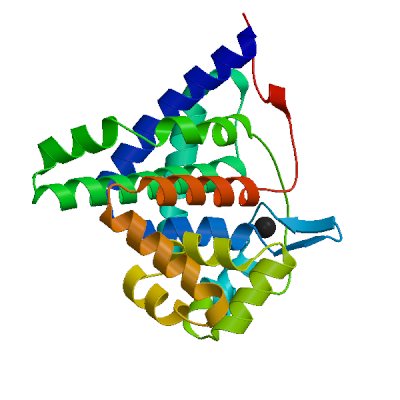

Supplement: Supplementary file 1 [file genes-12-00334-s001.zip › Supplementary file 2/Supplementary file 2/APX4/model/01/01.png]

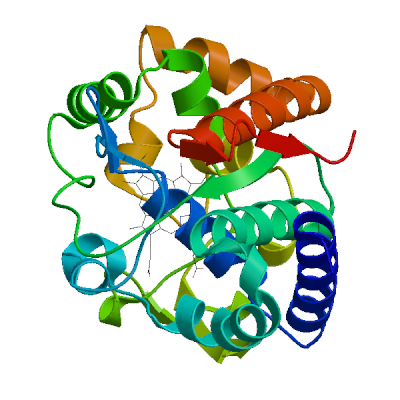

Supplement: Supplementary file 1 [file genes-12-00334-s001.zip › Supplementary file 2/Supplementary file 2/APX5/model/01/01.png]

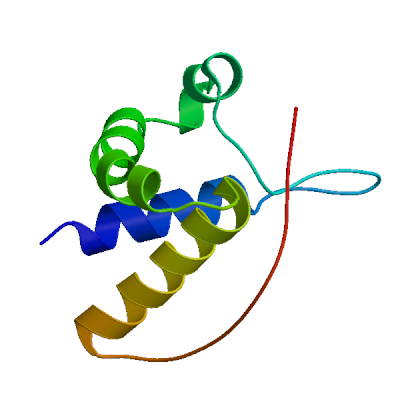

Supplement: Supplementary file 1 [file genes-12-00334-s001.zip › Supplementary file 2/Supplementary file 2/APX6/model/01/01.png]

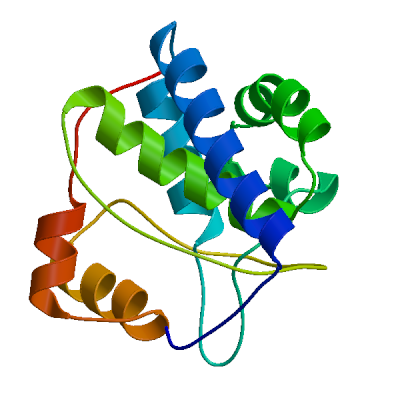

Supplement: Supplementary file 1 [file genes-12-00334-s001.zip › Supplementary file 2/Supplementary file 2/APX7/model/01/01.png]

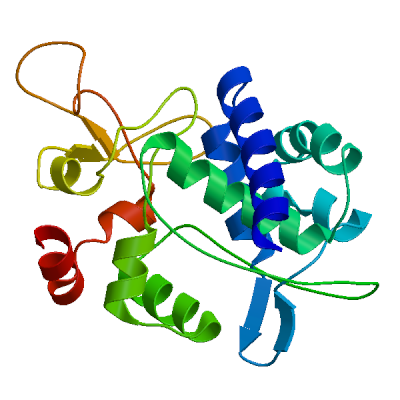

Supplement: Supplementary file 1 [file genes-12-00334-s001.zip › Supplementary file 2/Supplementary file 2/APX7/model/02/02.png]

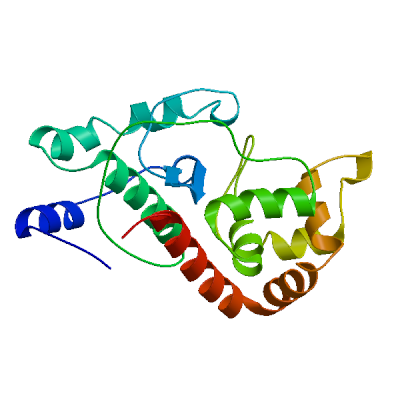

Supplement: Supplementary file 1 [file genes-12-00334-s001.zip › Supplementary file 2/Supplementary file 2/APX8/model/01/01.png]

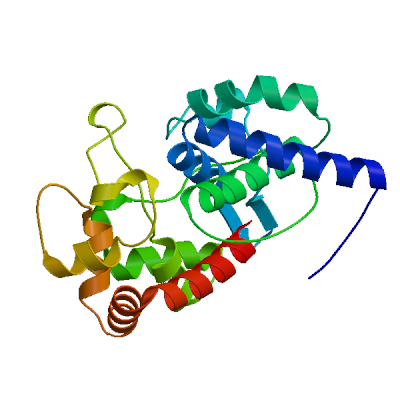

Supplement: Supplementary file 1 [file genes-12-00334-s001.zip › Supplementary file 2/Supplementary file 2/APX9/model/01/01.png]
